# Establishment of DNA-DNA Interactions by the Cohesin Ring

**DOI:** 10.1016/j.cell.2017.12.021

**Published:** 2018-01-25

**Authors:** Yasuto Murayama, Catarina P. Samora, Yumiko Kurokawa, Hiroshi Iwasaki, Frank Uhlmann

**Affiliations:** 1 Institute of Innovative Research, Tokyo Institute of Technology, 2-12-1 Ookayama, Meguro-ku, Tokyo 152-8550, Japan; 2 Chromosome Segregation Laboratory, The Francis Crick Institute, 1 Midland Road, London NW1 1AT, UK; 3 Education Academy of Computational Life Sciences, Tokyo Institute of Technology, 2-12-1 Ookayama, Meguro-ku, Tokyo 152-8550, Japan

**Keywords:** Cohesin, SMC complexes, sister chromatid cohesion, second-DNA capture, *S. pombe*

## Abstract

The ring-shaped structural maintenance of chromosome (SMC) complexes are multi-subunit ATPases that topologically encircle DNA. SMC rings make vital contributions to numerous chromosomal functions, including mitotic chromosome condensation, sister chromatid cohesion, DNA repair, and transcriptional regulation. They are thought to do so by establishing interactions between more than one DNA. Here, we demonstrate DNA-DNA tethering by the purified fission yeast cohesin complex. DNA-bound cohesin efficiently and topologically captures a second DNA, but only if that is single-stranded DNA (ssDNA). Like initial double-stranded DNA (dsDNA) embrace, second ssDNA capture is ATP-dependent, and it strictly requires the cohesin loader complex. Second-ssDNA capture is relatively labile but is converted into stable dsDNA-dsDNA cohesion through DNA synthesis. Our study illustrates second-DNA capture by an SMC complex and provides a molecular model for the establishment of sister chromatid cohesion.

## Highlights

• The recombinant cohesin complex mediates DNA-DNA interactions
• Second-DNA capture by cohesin requires ssDNA
• DNA replication stabilizes second-strand capture
• Second-DNA capture at a replication fork could establish sister chromatid cohesion

## Graphical Abstract

**Figure:**
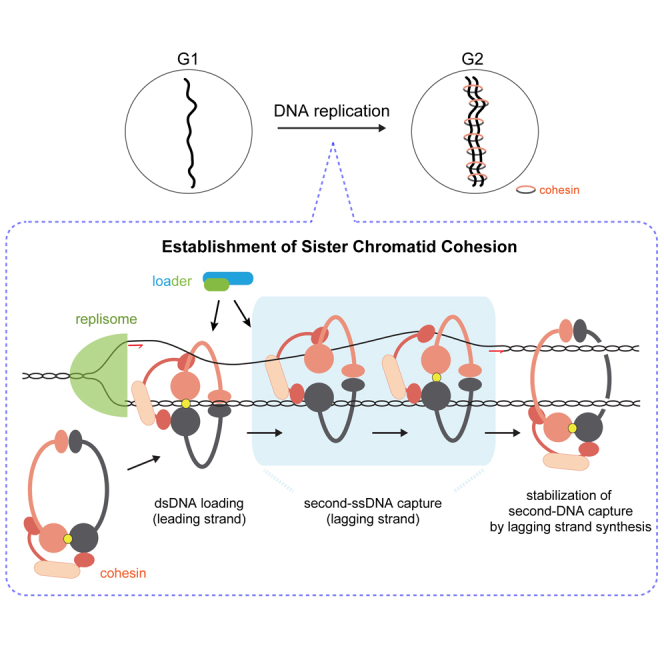

## Teaser

Cohesin sets up the initial steps of chromosome cohesion by anchoring single-stranded DNA along to a region of duplex DNA.

## Introduction

Chromosome organization by SMC complexes is vital for faithful chromosome segregation, DNA repair, and gene regulation. Deficiencies in SMC complexes and their regulators lead to a plethora of human malignancies, including developmental defects, infertility, and cancer (Liu and Krantz, 2008). SMC complexes are an evolutionarily conserved protein family that underpins genome organization in organisms, from bacteria to humans. Eukaryotes contain at least three essential family members: cohesin, condensin, and the Smc5-Smc6 complex. While cohesin and condensin are best known for their role in sister chromatid cohesion and chromosome condensation, respectively, the Smc5-Smc6 complex has been originally identified as a multi-subunit DNA repair complex with an essential but still incompletely understood role in chromosome segregation. A common feature of SMC complexes is that they bind to DNA by topological embrace (Hirano, 2016, Jeppsson et al., 2014, Nasmyth, 2011, Peters and Nishiyama, 2012, Uhlmann, 2016).

Topological DNA entrapment has been demonstrated for all three eukaryotic SMC family members, as well as a bacterial SMC complex. Furthermore, in the cases of cohesin and condensin, the topological nature of DNA binding has been suggested to be essential for SMC complex function (Cuylen et al., 2011, Haering et al., 2008, Kanno et al., 2015, Wilhelm et al., 2015). The cohesin complex maintains proximity between two replicated sister DNAs. Likewise, cohesin is thought to function in transcriptional regulation by bringing together two stretches of DNA within one chromatid to both form chromosome loops and engage in enhancer-promoter interactions (Hadjur et al., 2009). Chromosome condensation by condensin is equally thought to be mediated by bringing distant pieces of DNA into proximity, while stabilization of DNA-DNA contacts is also a plausible scenario for the function of the Smc5-Smc6 complex in DNA repair. Despite the explanatory power of DNA tethering, whether and how SMC complexes indeed directly mediate DNA-DNA interactions have remained open questions.

The cohesin ring is based on two SMC subunits: Smc1 and Smc3 (Psm1 and Psm3, fission yeast nomenclature is given in parenthesis). These are held together at a “hinge” dimerization interface from where long stretches of flexible coiled-coil reach out to ATP binding cassette (ABC) ATPase “head” domains. The head domains dimerize upon ATP binding, thus completing the ring. A kleisin subunit, Scc1 (Rad21), bridges the SMC heads to double up and reinforce their interaction. The kleisin also recruits two HEAT repeat subunits, Scc3 (Psc3) and Pds5, that regulate cohesin association with and dissociation from chromatin (Tanaka et al., 2001, Tomonaga et al., 2000). The cohesin ring is loaded onto chromatin well before DNA replication with the aid of a cohesin loader complex, a heterodimer of Scc2 and Scc4 (Mis4 and Ssl3) (Bernard et al., 2006, Furuya et al., 1998). The cohesin loader has been suggested to stabilize a cohesin conformation that exposes two DNA sensory lysines on the Smc3 ATPase head. Following contact with DNA and fueled by ATP hydrolysis and ATP rebinding, two interlocking gates between the ATPase heads and between Smc3 and the kleisin sequentially open to allow DNA entry (Murayama and Uhlmann, 2015).

DNA inside the cohesin ring can also engage the DNA sensory lysines and thus initiate passage through the same two interlocking gates, now leading to DNA exit. The exit reaction is facilitated by Pds5 together with a sub-stoichiometric cohesin subunit, Wapl. This leads to a dynamic binding and dissociation cycle of cohesin on chromosomes (Feytout et al., 2011, Kueng et al., 2006, Lopez-Serra et al., 2013). During DNA replication, the replication-fork-associated acetyltransferase Eco1 (Eso1) acetylates the two lysines. This stops further DNA passage through the cohesin gates and thereby leads to stable DNA entrapment within the cohesin ring, a prerequisite for stable sister chromatid cohesion (Murayama and Uhlmann, 2015, Rolef Ben-Shahar et al., 2008, Unal et al., 2008).

Following DNA replication, acetylated cohesin rings not only stably entrap one DNA, but they also hold together two sister chromatids. How cohesin achieves this tethering is not yet known. Protein crosslinking experiments have suggested that one cohesin ring embraces both sister chromatids. Alternatively, it has been proposed that two cohesin rings, holding one sister each, engage with each other or, in certain cases, with other chromatin components. Other models include the possibility that two cohesin rings fuse to create a double-sized ring that accommodates sister chromatids (Haering et al., 2008, Zhang et al., 2008). These various models make different predictions as to how cohesin achieves DNA-DNA tethering. If one cohesin ring embraces both sisters, this could be achieved if the replisome passes through DNA-bound cohesin rings. Alternatively, cohesin could sequentially entrap both sister DNAs as they lie close to each other in the wake of the replication fork (Lengronne et al., 2006).

Using purified fission yeast cohesin and its cohesin loader, we have previously reconstituted topological DNA entry into, as well as exit out of, the cohesin ring (Murayama and Uhlmann, 2014, Murayama and Uhlmann, 2015). While we have used double-stranded DNA (dsDNA) as substrate in these reactions, a bacterial SMC complex also binds single-stranded DNA (ssDNA) (Niki and Yano, 2016). We have now developed our assay system to ask whether DNA-bound cohesin can perform “second-DNA capture.” We find that this is indeed the case, but only if the second DNA is single stranded. Once second ssDNA capture is followed by DNA synthesis, cohesin stably and topologically entraps two dsDNAs. These results provide a molecular model of how cohesin engages in sister chromatid cohesion and maybe other chromosomal interactions.

## Results

### Second-DNA Capture by the Cohesin Ring

**Figure 1:**
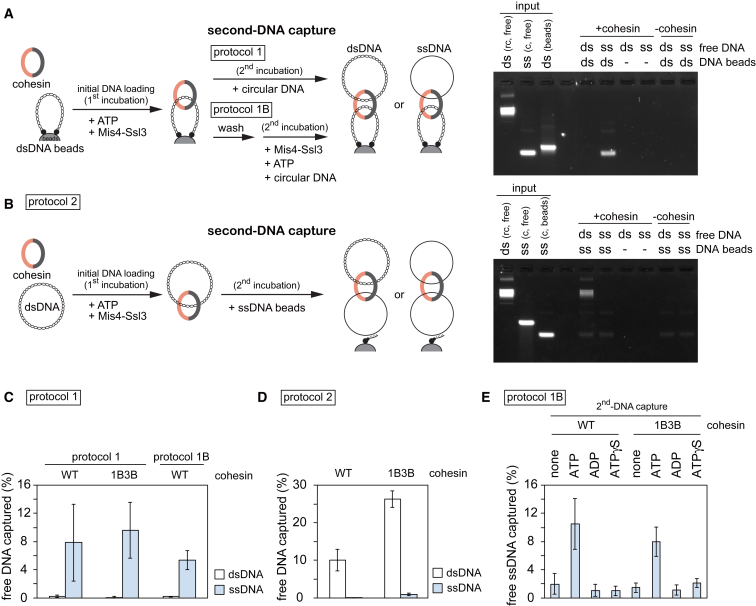
Second-DNA Capture by the Fission Yeast Cohesin Ring (A) Schematic of the second-DNA capture assay (protocol 1) and a gel image showing input and recovered DNA from the assay performed with the indicated substrates. ds, dsDNA; ss, ssDNA; rc, relaxed circular; c, circular. All reactions were carried out in the presence of ATP and an ATP regenerating system. 16.7% or 25% of input free dsDNA or ssDNA are shown. (B) Schematic of the second-DNA capture assay (protocol 2) and a representative gel image. 25% of input DNA is shown. (C and D) Quantification of the assays in (A) and (B), respectively, performed with WT and 1B3B cohesin. The means and standard deviations from three independent experiments are shown. (E) Quantification of second-DNA capture, using protocol 1B, in the absence or presence of the indicated adenosine derivatives. The means and standard deviations from three independent experiments are shown. See also Figure S1 for gel images that include supernatant fractions, reactions using 1B3B cohesin, titration of components, ATP competition, and an assay using ΦX174 ssDNA.

To test if, following initial DNA loading, cohesin can embrace a second DNA, we set up two types of *in vitro* second-DNA capture assays. In protocol 1, a dsDNA fragment, biotinylated at both ends, was bound to streptavidin magnetic beads to prepare a closed DNA topology (dsDNA beads, Figure 1A). Cohesin was incubated with these dsDNA beads in the presence of ATP and the cohesin loader, then free circular DNA was added to the same reaction for further incubation. dsDNA beads were sedimented and washed in a high-salt buffer, and captured circular DNA was analyzed by agarose gel electrophoresis (protocol 1). In protocol 2, a biotinylated oligonucleotide was annealed to circular ssDNA and immobilized to streptavidin magnetic beads (ssDNA beads, Figure 1B). Protocol 2 reactions started with initial cohesin loading onto free circular dsDNA in solution followed by transfer of the reaction onto the ssDNA beads. The captured DNA was eluted in both cases using protease K treatment in the presence of sodium dodecyl sulfate. Note that the biotin-streptavidin interaction is largely resistant to these elution conditions, resulting in no or only little elution of the biotinylated DNAs.

**Figure S1:**
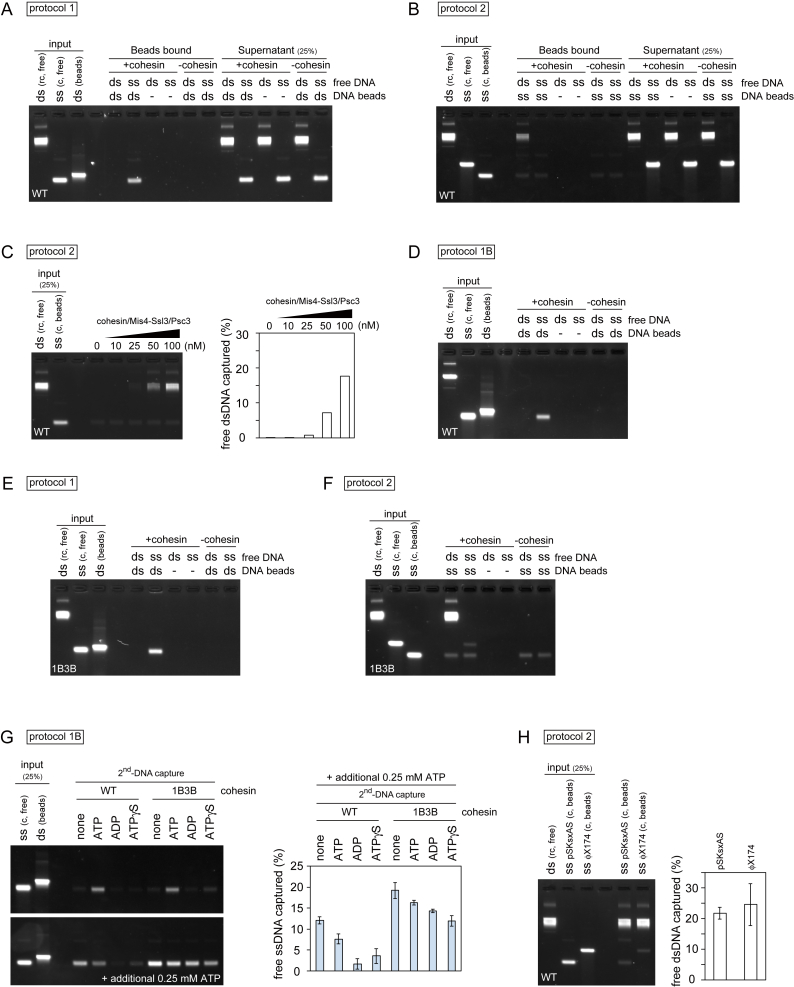
ssDNA, but not dsDNA, Is a Substrate for Second-Strand Capture, Related to Figure 1 (A and B) Extended gel images of Figures 1A and 1B, showing both beads bound and supernatant fractions. 25% of the supernatant fractions are shown. (C) Protocol 2 reactions were carried out using the indicated protein concentrations (each of cohesin, Mis4-Ssl3 and Psc3). The gel image and quantification of captured dsDNA are shown. (D) A typical gel image of inputs and products of a second-DNA capture reaction following protocol 1B, performed with WT cohesin. (E) Gel images showing second-DNA capture by 1B3B cohesin, containing Walker B mutations in both Psm1 and Psm3, using a protocol 1 reaction. (F) As (E), but the reaction followed protocol 2. (G) Competition of ATP with ADP or non-hydrolyzable ATP-γ−S. As in Figure 1E (top gel image), but an additional reaction was performed in which 0.25 mM of ATP was present in all reactions that were then supplemented by additional nucleotides. The ability of ADP and ATP-γ−S to compete with ATP demonstrates that both nucleotides are able to bind cohesin, but that their hydrolysis is required for second-DNA capture. (H) Cohesin mediates second-DNA capture irrespective of sequence homology. Protocol 2 reactions were carried out using pSKsxAS ssDNA (partially homologous to the free dsDNA substrate) or ΦX174 virion ssDNA (of unrelated sequence) as substrates. The graph presents means and standard deviations from three independent experiments.

In protocol 1, despite our intensive trials, cohesin that had been loaded onto immobilized dsDNA could not capture any detectable free circular dsDNA. However, if circular ssDNA was added, approximately 8% of the input-free ssDNA was retrieved under the same reaction conditions (Figures 1A, 1C, and S1A). Free ssDNA capture depended on cohesin and the presence of dsDNA on the beads, suggesting that cohesin tethered both DNAs. In protocol 2, up to 10% of the added free dsDNA was recovered on ssDNA beads in a cohesin and cohesin loader concentration-dependent manner (Figures 1B, 1D, S1B, and S1C). Following cohesin loading onto free dsDNA, approximately 25% of the input DNA is bound by cohesin under these conditions (Murayama and Uhlmann 2014). Assuming that cohesin-bound DNA is the substrate for second-DNA capture, approximately 40% of these were recovered on the ssDNA beads. If the first incubation in protocol 2 included ssDNA and cohesin, no ssDNA was captured on ssDNA beads (Figures 1B and 1D), suggesting a requirement for one dsDNA and one ssDNA to achieve second-DNA capture.

### Characterization of Second-ssDNA Capture

In both of the above protocols, the second DNA was added to reactions containing cohesin loaded onto the first DNA, as well as free cohesin. To test whether free cohesin is required for second-DNA capture, we modified protocol 1. Cohesin was again loaded onto dsDNA beads, but following the loading reaction, free cohesin and other components from the loading reaction were removed by a high-salt wash. Then, free DNA and cohesin loader, but no additional cohesin, were added (Figure 1A, protocol 1B). Free ssDNA, but not dsDNA, was retrieved as efficiently as before (Figures 1C and S1D). These results suggest that cohesin rings that already entrap dsDNA are able to capture a second ssDNA.

We previously found that ATP hydrolysis-dependent cohesin loading onto DNA is only mildly affected by Psm1 and Psm3 ATPase Walker B motif mutations (1B3B), which slow the rate of ATP hydrolysis. In contrast, cohesin unloading from DNA is greatly reduced by these mutations, effectively stabilizing cohesin on DNA (Murayama and Uhlmann, 2015). When we performed second-strand capture using 1B3B cohesin, we observed a markedly increased capture efficiency in both experimental protocols 1 and 2 (Figures 1C, 1D, S1E, and S1F). This is a first indication that captured ssDNA is not very stably bound by cohesin and that reducing the rate of ATP hydrolysis increases its retention.

Initial dsDNA loading of cohesin requires ATP hydrolysis (Murayama and Uhlmann, 2014), so we wondered if this is also the case for second-DNA capture. Using protocol 1B, we omitted ATP in the second-strand capture reaction or replaced it with ADP or non-hydrolyzable ATP-γ-S. Only ATP supported DNA tethering by either wild-type (WT) or 1B3B cohesin (Figure 1E). Furthermore, addition of ADP or ATP-γ-S competed with present ATP and impeded ssDNA capture (Figure S1G). This suggests that second-DNA capture by cohesin depends on ATP and requires its hydrolysis.

Our ssDNA and dsDNA substrates derive from related pBluescript plasmid backbones. To rule out that annealing of homologous DNA sequences plays a role in the DNA tethering seen in our assays, we performed second-DNA capture using unrelated ΦX174 ssDNA as a substrate. In a protocol 2 experiment, both ssDNAs retrieved similar amounts of dsDNA (Figure S1H), suggesting that second-DNA capture is independent of sequence homology.

### Second-DNA Capture Is Topological in Nature

**Figure 2:**
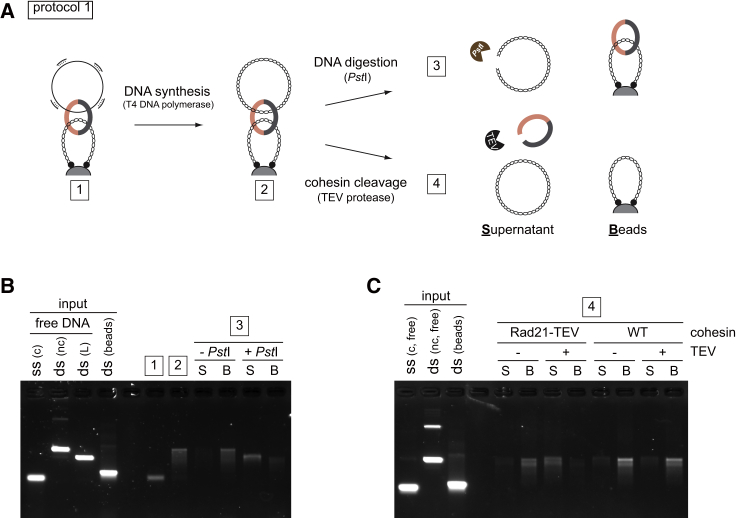
Second-DNA Capture Results in Topological Embrace (A–C) (A) Schematic and (B) gel images of the DNA-release experiments by restriction enzyme cleavage of captured DNA or (C) cohesin cleavage by TEV protease following ssDNA to dsDNA conversion. The gel images show input and recovered DNA at each of the indicated steps. nc, nicked circular; L, linear; WT, wild-type cohesin; Rad21-TEV, TEV protease cleavable cohesin; TEV, TEV protease; S, supernatant; B, beads bound fraction. See also Figure S2 for further experiments that confirm the topological nature of second-DNA capture.

**Figure S2:**
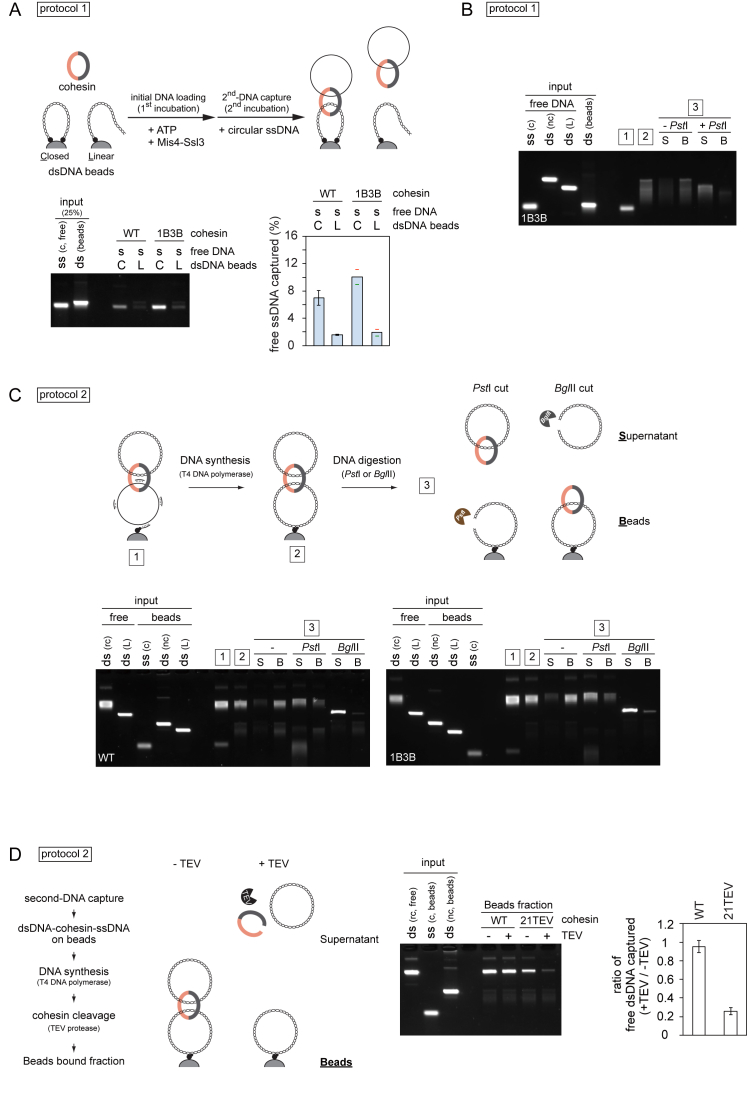
Second-DNA Capture is Topological in Nature, Related to Figure 2 (A) Cohesin must topologically embrace dsDNA to mediate second-DNA capture. Protocol 1 reactions were carried out with ‘closed’ (C) or ‘linear’ (L) topology dsDNA beads. The gel image and graph show recovery of free ssDNA. The graph shows means and standard deviation from three independent experiments (WT cohesin) or the range of recovered ssDNA from two independent experiments (1B3B cohesin). (B) A DNA release experiment as shown in Figure 2A was carried out using 1B3B cohesin. (C) Schematic of a DNA release experiment following protocol 2 s DNA capture. The ssDNA substrate was converted to dsDNA by DNA synthesis following capture. Then either of the two circular DNAs was digested with unique restriction enzymes, PstI (DNA beads) or BglII (free dsDNA). Recovered DNAs at the indicated stages of the experiment were analyzed by agarose gel electrophoresis. Input, bead bound (B) and supernatant (S) fractions are shown. (D) TEV cleavage of cohesin following second-DNA capture using protocol 2. The gel shows a representative image of input and recovered DNA, using WT and TEV cleavable (21TEV) cohesin, without or with TEV protease (TEV) treatment. After TEV protease treatment, the beads were washed with high salt buffer and recovered DNA was analyzed. The graph depicts the means and standard deviations from three independent experiments.

To investigate whether both dsDNA and ssDNA are topologically entrapped by cohesin, we initially carried out a protocol 1 reaction using dsDNA with only one biotinylated end, resulting in “linear dsDNA beads.” These linear dsDNA beads did not support second-strand capture (Figure S2A), suggesting that cohesin must be topologically loaded onto dsDNA to entrap a second ssDNA. We next wanted to know if cohesin also topologically embraces the second ssDNA. We carried out ssDNA capture on dsDNA beads using circular ssDNA to which short oligonucleotides were annealed. After washing, the captured ssDNA was converted to dsDNA using T4 DNA polymerase. Now, the second captured DNA was linearized by restriction enzyme treatment. This caused release of the captured DNA from the cohesin-bound dsDNA beads (Figures 2A and 2B), suggesting that it was bound by topological embrace. The same was observed in case of 1B3B cohesin (Figure S2B). Note that oligonucleotides were annealed at about 1 kb intervals; thus, cohesin appears to accommodate DNA polymerase progression at least within this distance. We subsequently also used a protocol 2 reaction to confirm that both the first and second DNAs are topologically entrapped by cohesin (Figure S2C).

Cleavage of cohesin’s kleisin subunit by the protease separase triggers sister chromatid separation at anaphase onset (Uhlmann et al., 2000). We therefore wanted to know whether Rad21 cleavage disrupts the *in vitro* DNA-DNA interaction mediated by our purified cohesin complex. To recapitulate Rad21 cleavage *in vitro*, we used a Rad21 variant in which one of the two separase cleavage sites is replaced with a TEV protease recognition site. We performed second-DNA capture using protocol 1 followed by dsDNA synthesis. Addition of TEV protease led to release of captured dsDNA only when TEV cleavable cohesin was used (Figures 2A and 2C). We obtained the same results when using a protocol 2 reaction (Figure S2D). This suggests that cohesin mediates cohesion between two DNAs *in vitro* in a manner that resembles sister chromatid cohesion *in vivo*.

### ssDNA Embrace Is Facile but Labile

**Figure 3:**
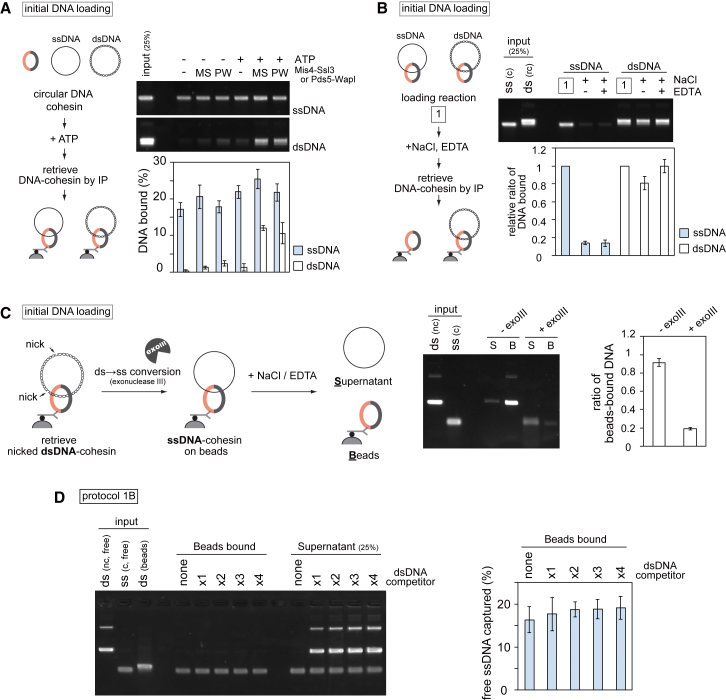
Topological but Labile ssDNA Embrace by the Cohesin Ring (A) Gel images and quantification of cohesin-loading assays using ssDNA or dsDNA as the substrate. Mis4-Ssl3 (MS) or Pds5-Wapl (PW) were added in the presence or absence of ATP. The graph shows the means and standard deviation from three independent experiments. (B) Following loading, the recovered material was challenged with NaCl and EDTA. The gel image and graph show DNA recovery at the indicated stages of the experiment. Means and standard deviations from three independent experiments are given. (C) Schematic and outcome of the dsDNA-to-ssDNA conversion experiment using *E. coli* exonuclease III (exoIII). Supernatant (S) and beads-bound (B) fractions were analyzed after the NaCl and EDTA chase. The graph indicates means and standard deviations from three independent experiments. (D) Specificity of second-ssDNA capture. Gel images and quantification of the protocol 1B second-DNA capture experiments in the presence of indicated ratio of nicked circular dsDNA competitor. The graph shows the means and standard deviation from three independent experiments. See also Figure S3, showing ssDNA stimulation of the cohesin ATPase and a control that released ssDNA remains circular.

**Figure S3:**
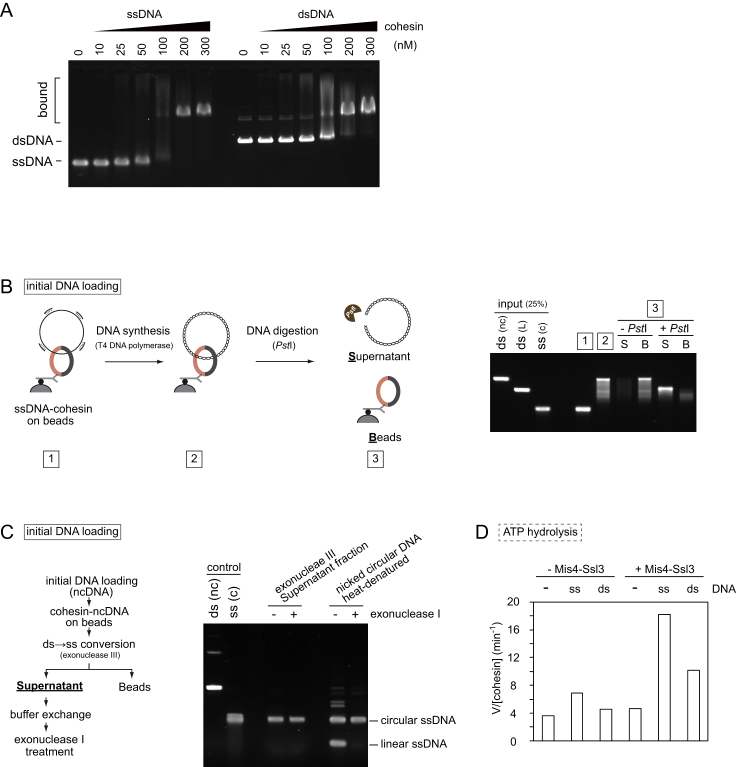
Further Characterization of ssDNA-Cohesin Interactions, Related to Figure 3 (A) ssDNA and dsDNA binding of cohesin were analyzed by electrophoretic mobility shift experiments using the indicated cohesin concentrations and single or double stranded pBluescript as the substrate. (B) ssDNA is topologically entrapped by cohesin. A schematic of the DNA-release experiment following ssDNA to dsDNA conversion is shown together with a gel image of the input and recovered DNAs at the indicated stages. (C) Cohesin releases circular ssDNA. The released DNA from cohesin following dsDNA to ssDNA conversion, as shown in Figure 3D, was treated with *E. coli* exonuclease I that digests linear but not circular ssDNA. No detectable digestion was observed, suggesting that released ssDNA remained circular. As a control for the effectiveness of exonuclease I treatment, heat-denatured nicked circular DNA was treated with exonuclease I in the same way. Linear, but not circular ssDNA was readily digested under these conditions. Note that two nicks were present in the circular DNA before denaturation. This generated two linear ssDNA, only the longer one of which is visible on the gel. (D) ssDNA stimulates the cohesin ATPase. Mis4-Ssl3 and DNA-dependent ATP hydrolysis by cohesin was measured in the presence of indicated proteins and DNAs.

To understand why second-DNA capture requires ssDNA, we characterized cohesin loading onto ssDNA as a first substrate. Cohesin showed similar ATP-independent electrostatic affinity to either ssDNA or dsDNA as judged by an electrophoretic gel mobility shift assay (Figure S3A). We then compared circular ssDNA or dsDNA as substrates in cohesin loading assays. As previously observed, topological loading of cohesin onto dsDNA required the presence of ATP and either Mis4-Ssl3 or Pds5-Wapl to stimulate loading (Figure 3A) (Murayama and Uhlmann, 2015). In the case of ssDNA, ATP or Mis4-Ssl3 stimulated DNA binding only to a small degree, and efficient binding was observed even in their absence. This suggests that cohesin binds ssDNA readily and under less stringent control compared to dsDNA. At the same time, ssDNA binding appeared less stable. Cohesin-ssDNA complexes were sensitive to salt and EDTA treatment, leading to ssDNA loss. In contrast, once cohesin topologically encircles dsDNA, the association becomes resistant to these treatments (Figure 3B).

Despite its labile nature, cohesin binding to ssDNA was topological in nature. After retrieving cohesin-ssDNA complexes, we converted ssDNA to dsDNA by DNA synthesis. Linearization released the captured DNA, suggesting that it had been topologically bound (Figure S3B). A drawback of this experiment is that it did not directly report on the topological status of the ssDNA; topological embrace might have been a consequence of ssDNA-to-dsDNA conversion. We therefore prepared topologically bound ssDNA in an alternative way. We first carried out a dsDNA loading assay using nicked dsDNA as the substrate. Following topological loading, dsDNA was converted to ssDNA using *E. coli* exonuclease III treatment. Now, the cohesin-DNA complexes were again exposed to salt and EDTA. ssDNA was released from cohesin, while dsDNA from a reaction that omitted exonuclease treatment retained stable binding (Figure 3C). This confirms that topologically entrapped ssDNA is easily lost from the cohesin ring. As an additional control, we verified that the released ssDNA remained circular and resistant to exonuclease I treatment (Figure S3C).

While the addition of ATP increased ssDNA binding by cohesin only to a small degree, ssDNA was an efficient stimulator of the cohesin ATPase. Cohesin shows a low intrinsic level of ATP hydrolysis, which increases in the presence of both the cohesin loader Mis4-Ssl3 and dsDNA. ssDNA was equally if not more efficient at stimulating the cohesin ATPase (Figure S3D), suggesting that ssDNA engages with cohesin in a physiologically meaningful manner.

Having characterized the interaction of cohesin with ssDNA, we returned to study second ssDNA capture by cohesin bound to dsDNA beads (protocol 1B). Given comparable affinities of cohesin to ssDNA and dsDNA, we expected that free dsDNA might compete with second-ssDNA capture. However, an up to 4-fold excess of nicked circular dsDNA did not interfere with second-ssDNA capture (Figure 3D). This suggests that once cohesin embraces dsDNA, it gains specificity for targeting a second ssDNA.

### Establishment of Stable DNA-DNA Cohesion by ssDNA-to-dsDNA Conversion

**Figure 4:**
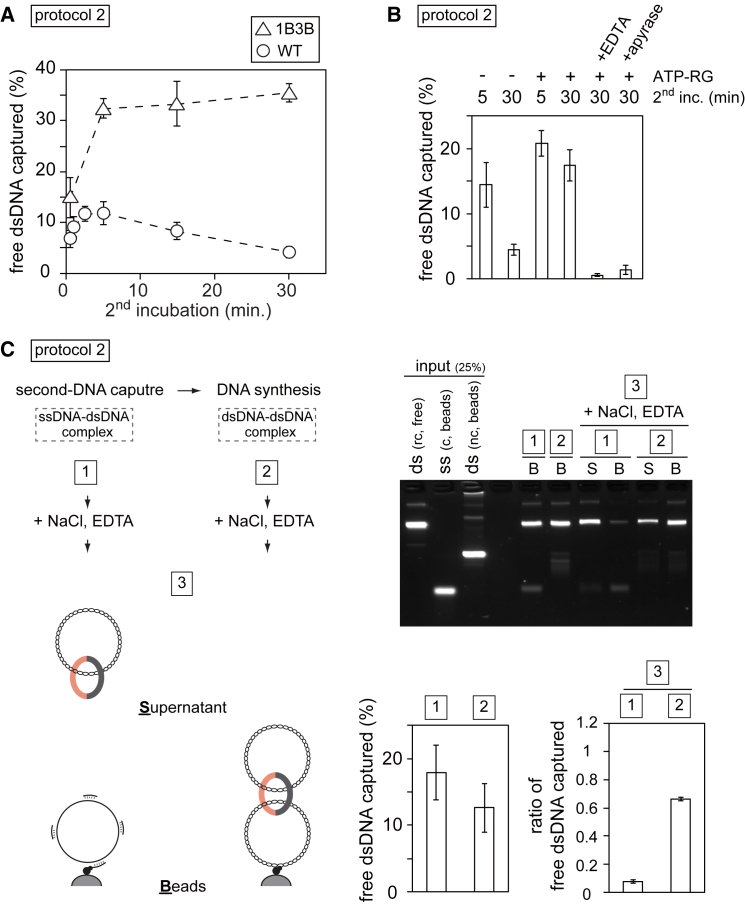
ssDNA-to-dsDNA Conversion Establishes Stable DNA-DNA Cohesion (A) Time course of second-DNA capture in a protocol 2 assay, comparing WT and 1B3B cohesin. The percentage of free dsDNA captured on ssDNA beads is plotted over time. (B) Same as (A), but the second-DNA capture incubation proceeded for 5 or 30 min in the absence or presence of an ATP-regenerating system (ATP-RG). The indicated additions were made 5 min into the second-DNA capture incubation. (C) Schematic of the experiment to convert ssDNA-to-dsDNA following second-DNA capture to test stabilization against NaCl and EDTA treatment. The gel image shows input and the recovered and released DNAs at the indicated stages of the experiment. The means and standard deviations from three independent experiments are shown in each panel.

Prompted by the labile nature of ssDNA entrapment, we more carefully monitored the kinetics of second-DNA capture. In a protocol 2 reaction, cohesin-bound dsDNA was rapidly captured as soon as it was added to ssDNA beads. Capture peaked at 5 min and gradually declined at later time points (Figure 4A). This is consistent with the observation that ssDNA tethering by cohesin is labile. In contrast, when we performed a similar time course experiment with 1B3B cohesin, the fraction of captured DNA increased over time until a plateau was reached after around 30 min. Because 1B3B cohesin hydrolyzes ATP at a reduced rate, we explored the possibility that an ATP-bound state was important for ssDNA retention. When we supplemented the second-DNA capture reaction with an ATP regenerating system, this led to increased DNA tethering by WT cohesin after 5 min and much improved stability over 30 min (Figure 4B). In contrast, adding apyrase that consumes ATP, or EDTA that prevents ATP action by chelating Mg^2+^ ions, abolished DNA tethering. This suggests that cohesin must bind ATP to retain topological ssDNA embrace.

Second-strand capture during sister chromatid cohesion establishment should result in enduring sister DNA linkages. The observation that dsDNA is bound more stably by cohesin than ssDNA suggests that ssDNA-to-dsDNA conversion might play a role in the establishment of stable DNA-DNA cohesion. We therefore tested whether dsDNA synthesis stabilizes DNA tethering. We started with a protocol 2 reaction by loading cohesin onto dsDNA. The product was added to primed ssDNA beads followed or not followed by DNA synthesis. We then tested the resistance of the cohesin-mediated DNA-DNA interaction to salt and EDTA by analyzing both beads-bound and supernatant fractions. Without DNA synthesis, over 90% of the captured DNA was released into the salt and EDTA supernatant (Figure 4C). In contrast, nearly 70% of captured DNA remained bound to beads following ssDNA-to-dsDNA conversion. This demonstrates that DNA synthesis stabilizes the resultant dsDNA-dsDNA linkages.

### Strict dsDNA-ssDNA Order of Second-DNA Capture

**Figure 5:**
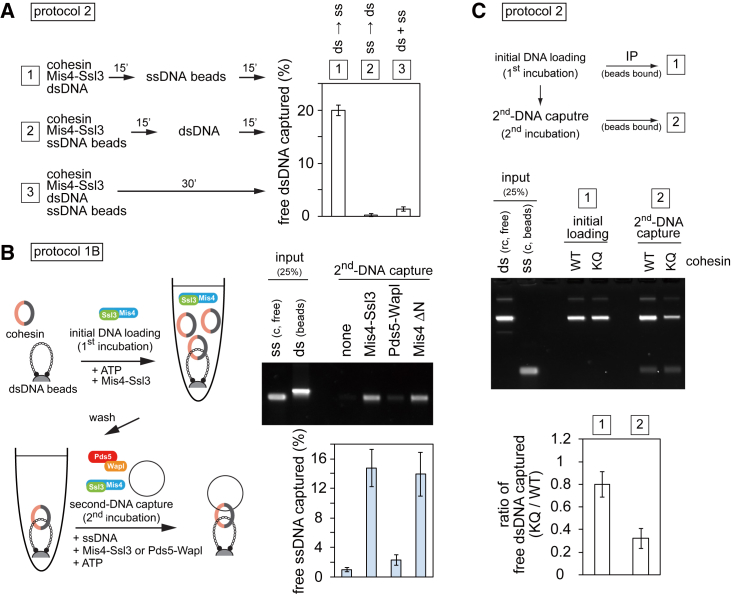
Acetyl-Acceptor Lysines and the Cohesin Loader Promote Second-DNA Capture (A) Protocol 2 experiments were carried out with the indicated order of additions, demonstrating a strong preference of reaction order during second-DNA capture. (B) Protocol 1B was used to test the ability of the indicated cohesin loading cofactors to promote second-DNA capture. Recovered DNA was analyzed by agarose gel electrophoresis and quantified. (C) WT and Psm3^K106Q^ (KQ) cohesin was used in a protocol 2 experiment. An aliquot was taken after the first dsDNA loading incubation to confirm comparable levels of loading by carrying out the reaction in low-salt condition (15 mM NaCl) before performing second-DNA capture on ssDNA beads followed by agarose gel electrophoresis and quantification. The means and standard deviations from three independent experiments are shown in each panel. See also Figure S4 for experiments that confirm the Mis4 requirement for second-DNA capture and the contribution of the acetyl-acceptor lysines to cohesin loading onto ssDNA.

During the course of our experiments, we noticed that second-DNA capture depended on the order in which cohesin binds to its two DNA substrates. This is demonstrated in an experiment in which we varied the order of reagent addition during second-DNA capture. In the original protocol 2, cohesin is loaded onto dsDNA in solution before the reaction is added to ssDNA beads (order 1), resulting in second-DNA capture (Figure 5A). In contrast, if cohesin and cohesin loader are first added to the ssDNA beads and dsDNA is added thereafter (order 2), no second-DNA capture was observed. When we simultaneously added all components to the ssDNA beads (order 3), second-DNA capture remained inefficient. This implies that cohesin must first load onto dsDNA to subsequently capture a ssDNA, a reaction order that cannot be reversed.

### Second-DNA Capture Depends on the Cohesin Loader

**Figure S4:**
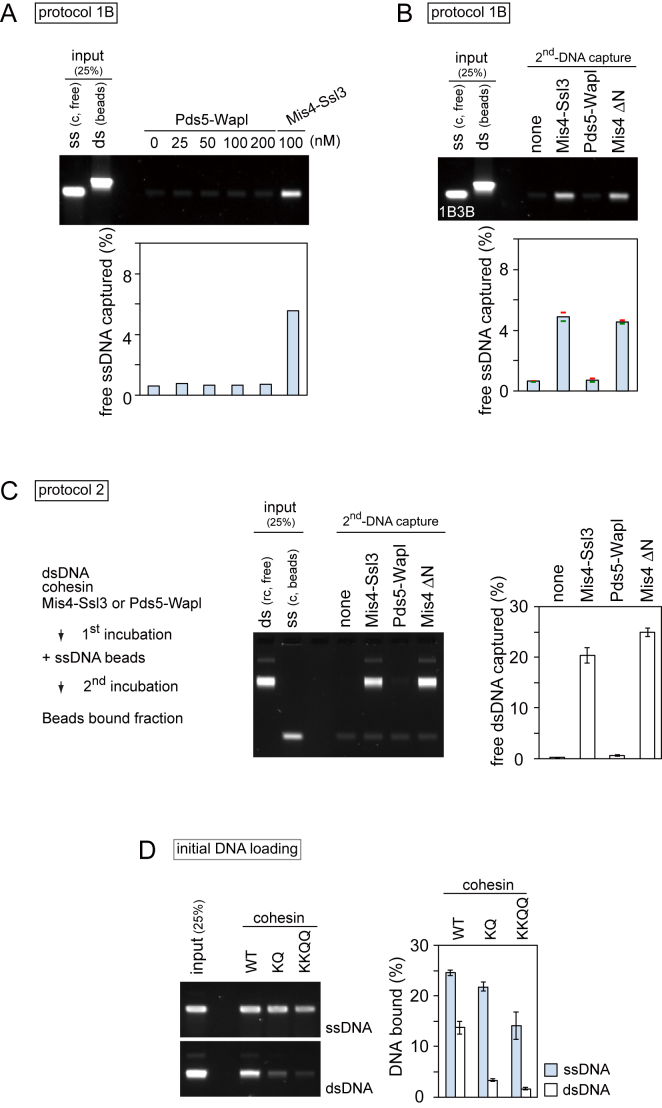
Mis4-Ssl3, but not Pds5-Wapl, Promote Second-DNA Capture, Related to Figure 5 (A) Protocol 1B reactions were initiated by cohesin and Mis4-Ssl3. Then the dsDNA beads were washed and increasing concentrations of Pds5-Wapl were included for second-DNA capture. A reaction in which Mis4-Ssl3 was added back is included for comparison. The graph shows quantification of recovered free ssDNA detected by agarose gel electrophoresis. (B) Protocol 1B reactions were carried out in the presence of the indicated loading cofactors as described in Figure 5B, but 1B3B cohesin was used. The graph shows means and the range of recovered ssDNA from two independent experiments. (C) Protocol 2 reactions were carried out in the presence of the indicated loading cofactors. The gel image shows recovery of free dsDNA on the ssDNA beads, the graph reports means and standard deviations from three independent experiments. (D) Acetyl-acceptor lysines on Psm3 contribute to ssDNA loading. DNA loading reactions were carried out with the indicated cohesin complexes using dsDNA or ssDNA as substrates. The graph shows means and standard deviations of the recovered DNA from three independent experiments.

To understand the mechanism of second-DNA capture, we further characterized its requirements. We used protocol 1B to load cohesin onto dsDNA beads in the presence of Mis4-Ssl3 followed by a high-salt wash to remove the cohesin loader. We then added free ssDNA with or without additional Mis4-Ssl3. This revealed that second-DNA capture strictly depended on the cohesin loader (Figure 5B). The *in vitro* dsDNA loading activity of the cohesin loader is contained within a C-terminal portion of Mis4 and does not require Ssl3 or the Mis4 N terminus (Mis4 ΔN) (Chao et al., 2015). Second-DNA capture was equally supported by Mis4 ΔN, suggesting that the cohesin loader plays a similar role during both first- and second-DNA capture. As an alternative to Mis4-Ssl3, *in vitro* cohesin loading onto dsDNA is promoted by Pds5-Wapl (Figure 3A) (Murayama and Uhlmann, 2015). In contrast, Pds5-Wapl did not support second-DNA capture even at increased concentrations (Figures 5B and S4A). Similar results were obtained using 1B3B cohesin, as well as using a protocol 2 assay (Figures S4B and S4C). This reveals that DNA-DNA tethering by cohesin firmly depends on the Mis4 cohesin loader.

### Acetyl-Acceptor Lysines on Psm3 Contribute to Second-DNA Capture

Two conserved lysines on the Psm3 ATPase head, which mediate DNA-stimulated ATP hydrolysis, are crucial for dsDNA entry into and exit out of the cohesin ring. Replacing both lysines with glutamines renders cohesin unable to load onto dsDNA, while replacing one lysine, Psm3^K106Q^, reduces dsDNA loading (Murayama and Uhlmann, 2015). These lysine replacements had a similar effect on the efficiency of ssDNA loading as a first substrate (Figure S4D). To test the impact on second-DNA capture, we prepared comparable amounts of WT and Psm3^K106Q^ cohesin-dsDNA complexes for use in a protocol 2 reaction (Figure 5C). Following their addition to ssDNA beads, DNA tethering by Psm3^K106Q^ cohesin was markedly reduced. This suggests a role for the acetyl-acceptor lysines, and therefore DNA-stimulated ATP hydrolysis, during second-DNA capture.

### ssDNA Binding Protein (RPA) Blocks Second-DNA Capture

**Figure 6:**
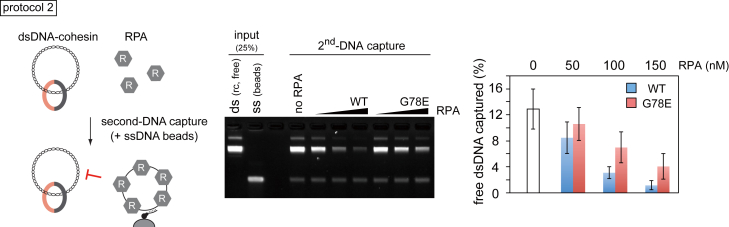
RPA Competes with Cohesin for Second-ssDNA Capture Schematic of the experiment in which cohesin was first loaded onto dsDNA, then increasing amounts of RPA (WT) or RPA containing Rfa1^G78E^ (G78E) were added before transfer onto ssDNA beads. The gel image shows input and recovered DNAs, and the graph shows means and standard deviations from three independent experiments. See also Figure S5 for a ssDNA binding assay comparing RPA and RPA containing Rfa1^G78E^.

**Figure S5:**
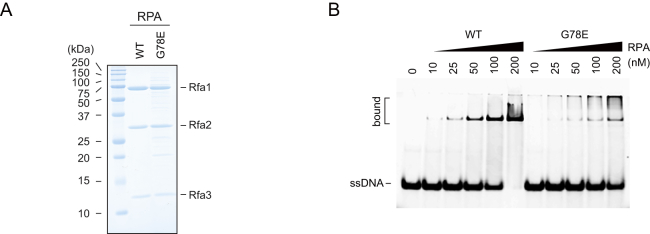
ssDNA Binding by RPA and RPA Containing Rfa1^G78E^, Related to Figure 6 (A) Purified hetero-trimeric RPA (WT) and RPA containing Rfa1^G78E^ (G78E) were analyzed by SDS-PAGE followed by Coomassie Blue staining. (B) ssDNA binding activity of WT and the mutant RPA complex was analyzed in an electrophoretic mobility shift experiment using single stranded (dT)_54_ as the substrate.

Our results so far suggest sequential dsDNA and ssDNA capture as a means by which cohesin establishes topological DNA interactions. However, free single-stranded DNA *in vivo* is thought to be covered by RPA, the heterotrimeric single-stranded DNA-binding protein (Fanning et al., 2006). We therefore investigated whether RPA interferes with second-DNA capture. We purified fission yeast RPA (Figure S5A) and included it in a competition assay using protocol 2. Cohesin was loaded onto dsDNA in solution, then increasing amounts of RPA were supplemented before both together were added to ssDNA beads. This revealed RPA concentration-dependent inhibition of second-DNA capture (Figure 6). Addition of 150 nM RPA in the reaction almost completely abolished second-DNA capture. This RPA concentration is sufficient to cover around half of all single-stranded sequences on the ssDNA beads, suggesting that a substantial stretch of free ssDNA is required for efficient second-DNA capture.

To address whether RPA inhibited second-DNA capture by ssDNA sequestration, we also purified RPA carrying a G78E mutation in the ssDNA-binding OB fold of its large Rfa1 subunit. This mutation was originally identified as a suppressor of a fission yeast condensin mutation and shows reduced ssDNA binding *in vitro* (Figures S5A and S5B) (Akai et al., 2011). RPA^G78E^ showed a reduced ability to inhibit second-DNA capture, compared to WT RPA (Figure 6). This confirms that RPA inhibits second-DNA capture by ssDNA sequestration.

### RPA Counteracts Sister Chromatid Cohesion Establishment *In Vivo*

**Figure S6:**
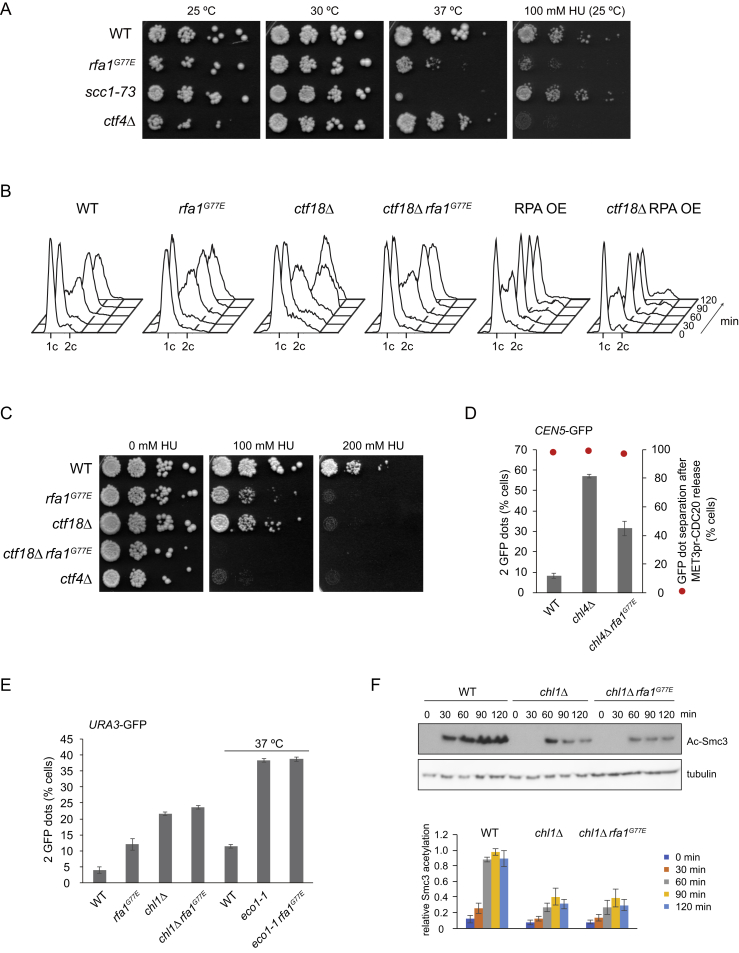
Additional Characterization of Budding Yeast *rfa1*^*G77E*^, Related to Figure 7 (A) Characterization of the budding yeast *rfa1*^*G77E*^ mutant. 10-fold serial dilutions of cultures of the indicated strains were spotted on YPD plates and grown at the indicated temperatures, or were spotted on YPD plates containing 100 mM HU that were incubated at 25°C. (B) Cell-cycle progression in synchronized cultures of the indicated strains from Figures 7A and 7B was monitored by FACS analysis of DNA content. (C) *rfa1*^*G77E*^ does not rescue the HU sensitive growth of *ctf18Δ* cells. 10-fold serial dilutions of the indicated budding yeast cell cultures were spotted on YPD plates containing the indicated concentrations of HU and incubated at 25°C. (D) Rescue of the centromeric *chl4Δ* cohesion defect by *rfa1*^*G77E*^. Sister chromatid cohesion close to centromere 5 (- 12.6 kb) was analyzed in strains of the indicated genotypes. Cells were arrested in mitosis by depleting Cdc20 under control of the *MET3* promoter by methionine addition. At least 100 cells were scored in each experiment. The graphs show means and standard deviations from 3 independent experiments. In parallel, metaphase-arrested cells from the experiment were released into anaphase by washing with and further growth in minimal medium lacking methionine to re-induce Cdc20 expression. The percentage of GFP-marked chromosomes that split 20 min after release into anaphase is shown. (E) *rfa1*^*G77E*^ does not suppress cohesion defects of *chl1Δ* or *eco1-1* cells. Sister chromatid cohesion was analyzed as Figure 7A. At least 100 cells were scored in each experiment reported. The graphs show means and standard deviations from 3 independent experiments. (F) *rfa1*^*G77E*^ does not rescue Smc3 acetylation in *chl1Δ* cells. Smc3 acetylation was analyzed in synchronized cultures from the indicated strains in the experiment shown in panel (E) by western blotting.

ssDNA is generated at the DNA replication fork as the DNA helicase unwinds the DNA duplex in preparation for DNA replication. On the leading strand, ssDNA is continuously converted into dsDNA. In contrast, ssDNA periodically persists on the lagging strand before Okazaki fragment priming and synthesis. The proximity of both DNAs at the fork might form a fitting substrate for sequential dsDNA and ssDNA capture to establish sister chromatid cohesion. If ssDNA plays a role in second strand capture *in vivo*, then RPA might impact on sister chromatid cohesion establishment. To explore this, we generated a budding yeast strain containing an *rfa1*^*G77E*^ mutation equivalent to fission yeast *rfa1*^*G78E*^. As in fission yeast, this mutation caused temperature-sensitive growth and sensitivity to hydroxyurea (HU) (Figure S6A) (Akai et al., 2011).

**Figure 7:**
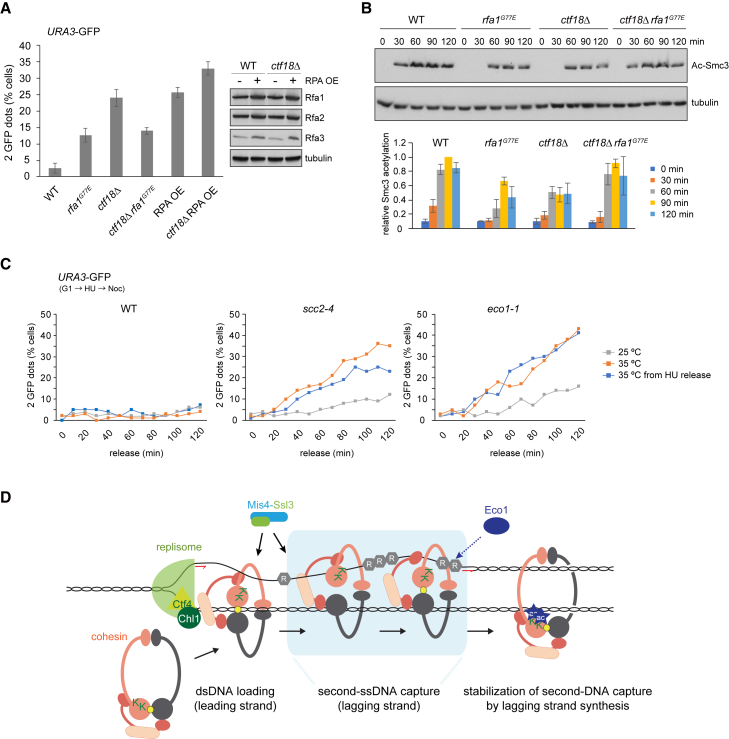
RPA Impacts on Sister Chromatid Cohesion Establishment *In Vivo* (A) Effect of the *rfa1*^*G77E*^ mutation or RPA overexpression (RPA OE) on sister chromatid cohesion in *ctf18Δ* cells. Cells were synchronized and arrested in mitosis by nocodazole treatment. Sister chromatid cohesion at the GFP-marked *URA3* locus was analyzed. Western blotting confirmed RPA overexpression. At least 100 cells were scored under each condition. The graph shows means and standard deviations from three independent experiments. (B) Smc3 acetylation was analyzed in synchronized cultures from the strains above by western blotting. The acetyl-Smc3 signal, normalized to tubulin and then to the WT signal at 90 min, was quantified in three independent repeats of the experiment. The means and standard deviations are shown. (C) The cohesin loader promotes sister chromatid cohesion establishment. Sister chromatid cohesion was monitored at indicated time points following release from G1 or HU under the indicated conditions and genotypes. (D) A model for the establishment of sister chromatid cohesion at the DNA replication fork. See the Discussion for details. See also Figure S6 for supporting genetic and cell-cycle analyses that explore the role of RPA in sister chromatid cohesion establishment.

To test whether reduced RPA affinity for ssDNA facilitates sister chromatid cohesion establishment, we employed cells lacking the Ctf18 cohesion establishment factor (Hanna et al., 2001). *ctf18Δ* cells are viable but show a marked sister chromatid cohesion defect (Figure 7A). The *rfa1*^*G77E*^ allele restored sister chromatid cohesion in *ctf18Δ* cells, suggesting that reduced competition for ssDNA indeed facilitates cohesion establishment. We note that *rfa1*^*G77E*^ by itself caused a mild cohesion defect and that restoration of cohesion in *ctf18Δ* cells was up to levels seen in *rfa1*^*G77E*^ cells. RPA might thus also make a positive contribution to cohesion establishment. While overt DNA replication was unaffected by the *rfa1*^*G77E*^ mutation (Figure S6B), smaller replication perturbations could alternatively have indirectly caused a cohesion defect. In contrast to sister chromatid cohesion, the HU-sensitive growth of *ctf18Δ* cells was not rescued by the *rfa1*^*G77E*^ mutation. Rather, *ctf18Δ* and *rfa1*^*G77E*^ caused synthetic lethality when exposed to HU (Figure S6C). This suggests that *rfa1*^*G77E*^ improves sister chromatid cohesion establishment specifically, but not other replication fork functions.

In contrast to reduced ssDNA affinity by *rfa1*^*G77E*^, increased RPA levels following overexpression of its three subunits (Yeeles et al., 2015) caused a strong sister chromatid cohesion defect, which worsened in the absence of Ctf18 (Figure 7A). This is consistent with a scenario in which cohesin and RPA compete for ssDNA during the establishment of sister chromatid cohesion.

As an additional readout for cohesion establishment, we monitored Smc3 acetylation, which is reduced by approximately half in the absence of Ctf18 (Figure 7B) (Borges et al., 2013). Acetylation was, to a lesser degree, reduced in *rfa1*^*G77E*^ cells, consistent with a positive role of RPA during cohesion establishment. In line with the rescue of sister chromatid cohesion, Smc3 acetylation was restored in *ctf18Δ rfa1*^*G77E*^ double-mutant cells. The interplay between Smc3 acetylation and RPA opens the possibility that cohesin acetylation is linked to second-ssDNA capture at the DNA replication fork.

We next investigated sister chromatid cohesion at centromeres, where the inner kinetochore component Chl4 plays a role in cohesion establishment (Fernius and Marston, 2009). The cohesion defect in *chl4Δ* cells, arrested in mitosis by depletion of the anaphase promoting complex activator Cdc20, was again improved by *rfa1*^*G77E*^ (Figure S6D), suggesting that ssDNA capture is important for cohesion establishment in this cohesin-rich region. Release into anaphase by Cdc20 re-induction caused sister chromatid splitting in both WT and *rfa1*^*G77E*^ cells (Figure S6D), ruling out the possibility that cohesion rescue by *rfa1*^*G77E*^ was an artifact of incomplete DNA replication.

Of other cohesion establishment mutants, the sister chromatid cohesion and Smc3 acetylation defects of cells lacking Chl1 were not rescued by *rfa1*^*G77E*^ (Figures S6E and S6F). A possible interpretation is that RPA and Chl1 act in one cohesion establishment pathway, parallel to Ctf18 (Borges et al., 2013). Use of ssDNA for cohesion establishment might depend on Chl1. The Eco1 acetyltransferase remained essential for sister chromatid cohesion in *rfa1*^*G77E*^ cells (Figure S6E), confirming that cohesion establishment relied on cohesin acetylation. Taken together, these observations lend support to the idea that ssDNA is a physiologically relevant substrate during sister chromatid cohesion establishment *in vivo*.

### The Role of the Cohesin Loader in Cohesion Establishment

We previously found that the budding yeast Scc2-Scc4 cohesin loader becomes dispensable for cell viability when cells are released from an HU arrest in early S phase. Under the same conditions, Eco1 remains essential for survival (Lengronne et al., 2006). This is unexpected if cohesion establishment involves cohesin-loader-dependent second-DNA capture. We therefore repeated the HU release experiment and directly monitored sister chromatid cohesion. This revealed a pronounced cohesion defect in *scc2-4* cells that were released at restrictive temperature from a short HU block (Figure 7C), consistent with a role of the cohesin loader in cohesion establishment. However, the cohesion defect was less than observed following loader inactivation already in G1, when no cohesin is loaded onto chromosomes. This suggests that the cohesin loader contributes to cohesion establishment but that a backup cohesion establishment pathway exists that is sufficient for survival following release from an HU block. Cohesion establishment depended on Eco1 at all times, and the cohesion defect was equally strong following inactivation of the *eco1-1* allele either in G1 or during release from the HU block.

## Discussion

Strong evidence has accumulated that cohesin holds sister chromatids together by topological embrace. Despite this simple model, the molecular architecture of such links and how cohesin is able to establish them remained poorly understood. Our biochemical study revealed a previously unknown biochemical activity of cohesin, which we refer to as second-DNA capture. Cohesin, once topologically loaded onto DNA, is able to entrap a second DNA. To our initial surprise, the second DNA strand must be single-stranded, and it requires ssDNA-to-dsDNA conversion by DNA synthesis to establish stable DNA-DNA cohesion.

### Second-DNA Capture by the Cohesin Ring

Second-ssDNA capture shares many features of the initial dsDNA loading reaction. Both reactions depend on the Psm3 acetyl-acceptor lysines that promote DNA-stimulated ATP hydrolysis, both require ATP hydrolysis, and both involve the Mis4 cohesin loader. We suggest that second-DNA capture is a repeat of initial dsDNA loading, with ssDNA transport through the two interlocking gates between the ATPase heads and the Psm3-kleisin interface (Murayama and Uhlmann, 2015). A striking difference from initial dsDNA loading is the requirement for ssDNA. We do not currently know the reason for this. ssDNA appears to enjoy relaxed entry and exit requirements compared to dsDNA, and this might aid second-DNA capture. The initially loaded dsDNA might occlude or obstruct parts of the cohesin ring involved in dsDNA entry. While dsDNA is stiff and highly charged, ssDNA is thinner, more flexible, and amphiphilic.

Retention of topologically trapped ssDNA by cohesin depended on ATP. Without ATP-dependent SMC head engagement, the Psm3-kleisin interface appeared unable to retain ssDNA. This is in contrast to dsDNA exit, which is efficiently blocked at the Psm3-kleisin gate and requires help from Pds5-Wapl to open (Beckouët et al., 2016, Murayama and Uhlmann, 2015). Which features allow ssDNA to slip through the Psm3-klesin gate and how these features might also facilitate second-DNA capture will be interesting to explore. Second-ssDNA capture showed exquisite dependence on the Mis4 cohesin loader. We speculate that a loader-assisted conformational change of the cohesin ring might be harder to achieve once a first dsDNA is contained within the ring. Therefore, Mis4 is more than a “cohesin loader.” Its most crucial role might be the establishment of DNA-DNA interactions.

Our current study leads to a biochemical model for how one cohesin ring sequentially entraps two DNAs. It does not directly describe the molecular structure of these DNA interactions. We cannot exclude that physical interactions between more than one cohesin ring contribute to DNA tethering in our assays. However, we consider this possibility unlikely. In our “protocol 1B,” a high-salt wash follows the initial cohesin loading onto dsDNA, which removes unbound protein. dsDNA-bound cohesin was subsequently able to topologically capture a second DNA without need for additional cohesin. This is hard to explain if a second DNA did not enter these cohesin rings (Figures 7D). The ability to generate DNA-cohesin-DNA complexes *in vitro* should aid with the clarification of their molecular architecture.

### Establishment of Sister Chromatid Cohesion

The characteristics of second-DNA capture *in vitro* unveiled an intriguing parallel to the DNA geometry at DNA replication forks. Replicated dsDNA on the leading strand is found next to periodically extended ssDNA on the lagging strand. Cohesin loaded onto the leading strand is aptly positioned to capture ssDNA on the lagging strand (Figure 7D). Shortly afterward, Okazaki fragment synthesis will convert cohesin’s fragile ssDNA embrace into stable sister chromatid cohesion. Cohesin can be detected at moving replication forks (Tittel-Elmer et al., 2012), consistent with the possibility of cohesin deposition during DNA replication.

The *rfa1*^*G77E*^ mutation and, by inference, an increased exposure of ssDNA improved cohesion defects in the absence of Ctf18 and in the absence of Chl4. Chl4 recruits Scc2-Scc4 and contributes to both cohesin loading and cohesion establishment at centromeres (Fernius et al., 2013). The greater availability of ssDNA might compensate for a reduced local Scc2-Scc4 concentration in *chl4Δ* cells. Consistently, HU treatment, which increases ssDNA exposure at forks, also restored sister chromatid cohesion in the absence of Chl4 (Fernius and Marston, 2009).

*rfa1*^*G77E*^ did not improve sister chromatid cohesion in cells lacking Chl1. This could be explained if Chl1 is part of the mechanism by which cohesin utilizes ssDNA. Chl1 is recruited to the replisome by Ctf4 and makes contact with cohesin during DNA replication (Samora et al., 2016). In this way, Chl1 might position cohesin favorably to capture both replicated DNAs. Chl1 is a DNA translocase, though its ATPase is dispensable for interaction with cohesin and, in budding yeast, makes only a minor contribution to sister chromatid cohesion. In mammalian cells, the Chl1 ATPase makes a greater contribution to cohesion establishment (Abe et al., 2016). As a translocase, Chl1 might clear RPA from ssDNA, or it could remove ssDNA secondary structures. The contribution of Chl1 to cohesion establishment remains to be further explored.

A mild cohesion defect due to the *rfa1*^*G77E*^ mutation suggests that RPA might also make a positive contribution to sister chromatid cohesion establishment. While we could not detect a positive role during second-DNA capture *in vitro*, using a broad range of concentrations (data not shown), RPA might contribute to cohesion establishment by an as-of-yet-unknown mechanism.

Establishment of sister chromatid cohesion comprises two steps. First, both sister DNAs have to be entrapped, then cohesin acetylation must stabilize the embrace. *rfa1*^*G77E*^ restored defective cohesin acetylation in the *ctf18Δ* background. Thus, in addition to facilitating second-DNA capture, increased ssDNA exposure enables cohesin acetylation, suggesting that the two events are linked. Cohesin acetylation requires ATP hydrolysis (Ladurner et al., 2014), which could form part of this link. How acetylation specifically occurs during second-ssDNA capture rather than first-dsDNA embrace will be important to address.

### Is Second-ssDNA Capture Conserved among SMC Complexes?

Sequential dsDNA-ssDNA capture appears tailored to entrap sister DNAs as they emerge from the replication fork. Might cohesin use a similar mechanism in other situations? In addition to replication forks, cohesin establishes sister chromatid cohesion at double-stranded DNA breaks (Ström et al., 2007, Unal et al., 2007). This requires the cohesin loader, but also the Mre11 exonuclease that resects DNA breaks to form ssDNA overhangs. Cohesin from the intact sister could target ssDNA on the damaged strand to reinforce cohesion around the break. In another role, cohesin establishes DNA loops within chromosomes that characterize interphase genome architecture, often between enhancers and gene promoters (Hadjur et al., 2009). Promoters are unwound in preparation for gene transcription, and ssDNA become accessible at these sites (Kouzine et al., 2013). Whether cohesin indeed targets ssDNA when establishing intra-chromosomal interactions is not known. Should ssDNA play a role in the formation of intra-chromosomal interactions, these might be less enduring than sister chromatid cohesion, owing to the fragile nature of ssDNA embrace.

Might ssDNA also be a substrate for other members of the SMC family? A bacterial SMC complex entraps both dsDNA and ssDNA (Niki and Yano, 2016). Fission yeast condensin, in turn, is recruited to actively transcribed genes in a manner that has been linked to the exposure of ssDNA (Sutani et al., 2015). Indeed, the fission yeast *rfa1*^*G78E*^ mutant was originally identified as a suppressor of a temperature-sensitive condensin mutation (Akai et al., 2011). One explanation of this genetic suppression is that RPA competes with condensin for ssDNA binding. The Smc5-Smc6 complex also shows affinity for ssDNA. It is recruited to DNA breaks and stalled replication forks where ssDNA is generated (Alt et al., 2017, Lindroos et al., 2006). Although a dedicated loader complex is known only for cohesin, the structural similarity of all SMC complexes suggest that they load onto DNA and might achieve second-DNA capture in similar ways. Our current study provides a first glimpse at how SMC complexes tether DNAs to mediate chromosome organization.

## STAR★Methods

### Key Resources Table

**Table:** 

REAGENT or RESOURCE	SOURCE	IDENTIFIER
**Antibodies**		

Mouse monoclonal anti-V5	Bio-Rad	Cat# MCA1360 RRID: AB_322378
Mouse monoclonal anti-HA (12CA5)	Sigma-Aldrich	Cat# 11583816001 RRID: AB_514505
Mouse monoclonal anti-E2a (5E11)	Abcam	Cat# ab977 RRID: AB_296610
Mouse monoclonal anti-histidine (HRP-conjugated)	MBL	Cat# D291-7 RRID: AB_10694870
Rabbit polyclonal anti-Psm1 (fission yeast)	BioAcademia	Cat# 63-137 RRID: AB_1056062
Rabbit polyclonal anti-Rad21 (fission yeast)	BioAcademia	Cat# 63-139
Rabbit polyclonal anti-Mis4 (fission yeast)	BioAcademia	N/A
Mouse monoclonal anti-acetyl Smc3 (budding yeast)	Gift from K. Shirahige	N/A
Rabbit polyclonal anti-RPA (budding yeast)	Agrisera	Cat# AS07 214 RRID:AB_1031803
Rat monoclonal anti-tubulin	BioRad	Cat# YOL1/34 RRID:AB_527345
Anti-mouse IgG (HRP-conjugated)	GE Healthcare	Cat# NA931 RRID:AB_772210
Anti-rabbit IgG (HRP-conjugated)	GE Healthcare	Cat# NA934 RRID:AB_772206
Anti-rat IgG (HRP-conjugated)	GE Healthcare	Cat# NA935 RRID:AB_772207

**Chemicals, Peptides, and Recombinant Proteins**		

Phenylmethylsulfonyl fluoride (PMSF)	Sigma-Aldrich	Cat# P7626
cOmplete EDTA-Free Protease Inhibitor Cocktail	Sigma-Aldrich	Cat# 04693132001
Phosphocreatine di(tris) salt	Sigma-Aldrich	Cat# P1937
ATP	Sigma-Aldrich	Cat# A2383
ADP	Sigma-Aldrich	Cat# A2754
ATPγS	Sigma-Aldrich	Cat# A1388
dNTP mixture	TaKaRa	Cat# 4030
Bio-Safe Coomassie Stain	Bio-Rad	Cat# 1610786
SYBR Gold Nucleic Acid Gel Stain	ThermoFisher	Cat# S11494
PureLink RNase A	ThermoFisher	Cat# 12091021
PreScission Protease	GE Healthcare	Cat# 27084301
AcTEV Protease	ThermoFisher	Cat# 12575015
Protease K	TaKaRa	Cat# 9034
PstI	Nippon Gene	Cat# 312-01171
BglII	TaKaRa	Cat# 1021A
*Xba*I	TaKaRa	Cat# 1093A
Nb*.Bss*SI	New England BioLabs	Cat# R0681S
Nt. BspQI	New England BioLabs	Cat# R0644S
T4 DNA polymerase	TaKaRa	Cat# 2040A
*E. coli* exonuclease I	TaKaRa	Cat# 2650A
*E. coli* exonuclease III	TaKaRa	Cat# 2170A
Tks Gflex DNA polymerase	TaKaRa	Cat# R060A
Creatine Kinase	Sigma-Aldrich	Cat# 10127566001
Fission yeast cohesin (Psm1-Psm3-Rad21-Psc3)	Murayama and Uhlmann, 2014	N/A
Fission yeast 1B3B cohesin (Psm1^E1161Q^-Psm3^E1128Q^-Rad21-Psc3)	Murayama and Uhlmann, 2015	N/A
Fission yeast KQ cohesin (Psm1-Psm3^K106^Q-Rad21-Psc3)	Murayama and Uhlmann, 2015	N/A
Fission yeast KKQQ cohesin (Psm1-Psm3^K105Q/K106Q^-Rad21-Psc3)	Murayama and Uhlmann, 2015	N/A
Fission yeast Rad21TEV cohesin (Psm1-Psm3-Rad21TEV-Psc3)	Murayama and Uhlmann, 2014	N/A
Fission yeast Mis4-Ssl3	Murayama and Uhlmann, 2014	N/A
Fission yeast Mis4ΔN	Chao et al., 2015	N/A
Fission yeast Psc3	Murayama and Uhlmann, 2014	N/A
Fission yeast Pds5	Murayama and Uhlmann, 2015	N/A
Fission yeast Wapl	Murayama and Uhlmann, 2015	N/A
Fission yeast RPA (Rfa1-Rfa2-Rfa3)	Akai et al., 2011	N/A
Fission yeast RPA^G78E^ (Rfa1^G78E^-Rfa2-Rfa3)	Akai et al., 2011	N/A
G418	Sigma-Aldrich	Cat# G8618
Hydroxyurea	Sigma-Aldrich	Cat# H8627
α-factor	Peptide Chemistry Laboratory, The Francis Crick institute	N/A
Nocodazole	Sigma-Aldrich	Cat# M1404

**Critical Commercial Assays**		

InFusion HD cloning kit	Clontech Laboratories	Cat# 639634
PrimeSTAR Max DNA Polymerase	TaKaRa	Cat# R045A
Human IgG-Agarose	Sigma-Aldrich	Cat# A6284-5ML
Glutathione Sepharose 4B	GE Healthcare	Cat# 17075601
TALON Metal Affinity Resin	Clontech Laboratories	Cat# 635501
HiTrap Heparin HP 1 mL	GE Healthcare	Cat# 17040601
HiTrap Capto Q 1 mL	GE Healthcare	Cat# 11001302
HiTrap Q HP 1 mL	GE Healthcare	Cat# 29051325
Superdex 200 Increase 10/300 GL	GE Healthcare	Cat# 28990944
Superose 6, 10/300 GL	GE Healthcare	Cat# 17517201
Amicon Ultra-4 centrifuge filter unit, 10 NMWL	MERCK MILLIPORE	Cat# UFC801024
MicroSpin S-400 HR columns	GE Healthcare	Cat# 27514001
MicroSpin G-25 columns	GE Healthcare	Cat# 27532501
Slide-A-Lyzer MINI Dialysis Devices, 3.5K MWCO, 0.1ml	ThermoFisher	Cat# 69550
Dynabeads M-280 Streptavidin	ThermoFisher	Cat#11206D
Dynabeads Protein A	ThermoFisher	Cat#10002D
ECL Prime Western Blotting Detection Regent	GE Healthcare	Cat# RPN2232
Hyperfilm ECL	GE Healthcare	Cat# 28906839

**Deposited Data**		

Mendeley Data dataset	This study	https://data.mendeley.com/datasets/wzmx278bj7/draft?a=60051c6f-7d50-42b8-abd8-67026ca46c18

**Experimental Models: Organisms/Strains**		

All *Saccharomyces cerevisiae* and *Schizosaccharomyces pombe* strains used in this study are listed in Table S1.	Lab stock and this study	N/A
*Escherichia coli*: BL21 (DE3) codonPlus RIPL chemical competent cells	Agilent Technologies	Cat# 230280

**Oligonucleotides**		

All oligonucleotides used for in biochemical reconstitution assays are listed in Table S2	Eurofins Genomics	N/A
Primer: R21 CGGGGGATCCACTAGTTCTAGAATCGGCAAGATT GTTCAAATGCAGCC	Eurofins Genomics	N/A
Primer: R2 TCACGCGAATGATATCttCCCTTTGTAGTTCCATTGACTGG	Eurofins Genomics	N/A
Primer: R3 GATATCATTCGCGTGATAATTGCAGAACC	Eurofins Genomics	N/A
Primer: R22 CCGCGGTGGCGGCCGCTCTAGAATGATGGTAATTTCATACTACT GAACGTAAATG	Eurofins Genomics	N/A
Primer: R4 ATTAACCCGGGGATCCGAGCTAACAAAGCCTTGGATAACTCATCG	Eurofins Genomics	N/A
Primer: R7 TAAACGAGCTCGAATTCTGTATCAAATAATCAAGTACTATTTAAT CTATGTAAC	Eurofins Genomics	N/A
Primer: R5 CGGATCCCCGGGTTAATTAACATC	Eurofins Genomics	N/A
Primer: R6 GAATTCGAGCTCGTTTAAACTGG	Eurofins Genomics	N/A

**Recombinant DNA**		

All phagemid ssDNA and plasmid DNA used for in biochemical reconstitution assays are listed in Table S3	This study, Murayama and Iwasaki, 2011, Murayama and Uhlmann, 2014	N/A
ΦX174 Virion DNA	New England BioLabs	Cat# N3023L
M13KO7 Helper Phage	New England BioLabs	Cat# N0315S
Plasmid: pFA6a-3HA-kanMX6	Van Driessche et al., 2005	N/A
Plasmid: pScRPA^G77E^-3HA::kanMX6	This study	N/A
Plasmid: pMis4-PA	Murayama and Uhlmann, 2014	N/A
Plasmid: pMis4ΔN-PA	Chao et al., 2015	N/A
Plasmid: pSsl3	Murayama and Uhlmann, 2014	N/A
Plasmid: pGEX-Psc3	Murayama and Uhlmann, 2014	N/A
Plasmid: pGEX-Wapl	Murayama and Uhlmann, 2015	N/A
Plasmid: pET15b-RPA	Akai et al., 2011	N/A
Plasmid: pET15b-RPA^G78E^	Akai et al., 2011	N/A

**Software and Algorithms**		

MultiGauge ver,3.2	Fuji Film	N/A
ImageQuant TL ver8.1	GE Healthcare	N/A

### Contact for Reagnet and Resource Sharing

Further information for resources and request should be directed to and will be fulfilled by the Lead Contact, Yasuto Murayama (ystmurayama@nig.ac.jp).

### Experimental Model and Subject Details

#### Yeast Strains

All budding yeast strains used in this study were of W303 background. Cells were cultured in YP medium (2% peptone and 1% yeast extract) containing 2% glucose (YPD) or in complete synthetic medium (0.67% yeast nitrogen base, BD Bioscience) lacking methionine and 2% glucose as the carbon source (Amberg et al., 2005). All strains were cultured at 25°C, except the temperature sensitive scc2-4 and *eco1-1* strains that were shifted to 35°C for inactivation. The *eco1-1* strain was grown at 23°C, but shifted to 37°C for Eco1 inactivation in the experiment shown in Figure S6. All fission yeast cells were cultured in EMM minimal medium supplemented with 30 μM thiamine and the indicated amino acids at 30°C (Forsburg and Rhind, 2006). Genotypes of all strains used are listed in Table S1.

#### Bacteria

Fission yeast Psc3, Wapl and RPA were expressed in the *E. coli* strain BL21-CodonPlus(DE3)-RIPL (Agilent Technologies). The genotype is: *E. coli B F*^*-*^ *ompT hsdS(r*_*B*_^*-*^ *m*_*B*_^*-*^*) dcm*^*+*^ *Tet*^*r*^ *gal λ(DE3) endA Hte [argU proL BBCamr] [argU ileY leuW Strep/Specr]*.

### Method Details

#### Plasmids Used for the Budding Yeast *rfa1*^*G77E*^ Mutant Strain Construction and Fission-Yeast Protein Expression

pScRPA^G77E^-3HA::kanMX6 was constructed as follows: The budding yeast *RFA1* gene containing 0.3 kb flanking regions was amplified with two primer sets [R21 and R2] or [R3 and R22] that encode the mutation, using genomic DNA from a W303 strain as the template. The resultant two DNA fragment were cloned into pBluescript SKII at its *Xba*I site using the InFusion HD cloning kit (Clontech Laboratories) to generate the *rfa1*^*G77E*^ mutant coding sequence. The plasmid was then amplified with a primers R4 and R7 and fused with DNA fragments containing the 3xHA tag and a G418 resistance gene (kanMX6) using the InFusion HD cloning kit. The 3HA-kanMX6 fragment was amplified with primers R5 and R6 from pFA6a-3HA-kanMX6 plasmid (Van Driessche et al., 2005). The other plasmids listed in the Key Resources Table (pMis4-PA, pMis4ΔN-PA, pSsl3, pGEX-Psc3., pGEX-Wapl, pET15b-RPA and pET15b-RPA^G78E^) were used for protein expression as previously described (Akai et al., 2011, Chao et al., 2015, Murayama and Uhlmann, 2014).

#### Protein Expression and Purification

All fission-yeast cohesin complexes (WT, Walker B mutant (Psm1 E1161Q Psm3 E1128Q, denoted as 1B3B cohesin), Psm3 acetyl-acceptor site mutants (K106Q, denoted as KQ and K105Q K106Q denoted KKQQ), TEV protease cleavable Rad21), Psc3, Mis4-Ssl3, Mis4ΔN (amino acids 192-1587), Pds5 and Wapl were expressed and purified as previously described (Chao et al., 2015, Murayama and Uhlmann, 2014, Murayama and Uhlmann, 2015).

In brief, the fission-yeast cohesin and Pds5 were expressed and purified from budding yeast as recombinant protein. Cells were grown in YP medium containing 2% raffinose and protein expression was induced by addition of galactose (2% final). Cells were disrupted in a cryogenic grinder under liquid nitrogen, the frozen cell powder was thawed on ice and the lysate was clarified by ultracentrifugation. The fission-yeast proteins were purified by sequential column chromatography (cohesin; IgG-agarose (Sigma), HiTrap Heparin HP (GE Healthcare) and Superose 6 Increase 10/300 GL (GE Healthcare), Pds5; IgG-agarose, HiTrap Capto Q (GE Healthcare) and Superdex 200 Increase 10/300 GL (GE Healthcare)). The cohesin loader complex was overexpressed and purified from fission yeast. Cells were grown in EMM lacking thiamine and disrupted in a cryogenic grinder under liquid nitrogen and the lysate was clarified by ultracentrifugation. The loader was purified by sequential column chromatography (IgG-agarose, HiTrap Heparin HP and Superdex 200 Increase 10/300 GL). Psc3 and Wapl were expressed in *E. coli* as GST-fusion proteins. Cells were disrupted by sonication and the lysate was clarified by ultracentrifugation. Proteins were purified by sequential column chromatography (Psc3; Glutathione Sepharose 4B (GE Healthcare), HiTrap Heparin HP and Superdex 200 Increase 10/300 GL, Wapl; Glutathione Sepharose 4B and HiTrap Heparin HP). The GST tag was removed by PreScission protease cleavage after elution from the Glutathione Sepharose 4B resin.

WT and G78E mutant heterotrimeric RPA complexes were purified as previously described with minor modifications (Akai et al., 2011). In brief, the fission yeast *rfa1+*, *rfa2+* and *rfa3+* cDNA were fused in one array, separated by internal ribosome entry sites in pET15b under the control of the T7 promoter. The Rfa1 subunit was fused to a 6 x histidine tag at the N terminus. The resultant plasmid was introduced into *E. coli* BL21 (DE3) codonplus RIPL. The cells were grown in Luria-Bertani (LB) medium containing 100 μg/ml ampicillin at 37°C until the optical density at 600 nm reached ∼0.3, then further grown at 28°C for 2 h. Isopropyl β-D-1-thiogalactopyranoside (IPTG) was added to a final concentration of 1 mM and growth continued at 18°C for 3.5 h. If not otherwise indicated, the following procedures were carried out at 4°C. The cells were harvested and resuspended in 5 volumes of H buffer (25 mM HEPES-KOH, pH 7.5, 10% glycerol) containing 500 mM NaCl, 3 mM imidazole and a protease inhibitor cocktail (Sigma). The cells were broken by sonication and the extract clarified by centrifugation at 100,000 x g for 30 min. The lysate was mixed with TALON resin (Clontech Laboratories Inc, 40 mL lysate per 1 mL slurry) and rocked for 2 h. The resin was packed into a column and washed with 50 bed volumes of H buffer containing 500 mM NaCl and 3 mM imidazole, followed by 10 bed volumes of H buffer containing 300 mM NaCl and 6 mM imidazole. Bound protein was eluted with 5 bed volumes of H buffer containing 300 mM NaCl and 200 mM imidazole. The eluate was diluted with R buffer (20 mM Tris-HCl pH 7.5, 10% glycerol, 0.5 mM dithiothreitol) to a final NaCl concentration of 100 mM and loaded onto a 1 mL HiTrap Sepharose Q column (GE Healthcare). Protein was eluted with a linear gradient from 0.1 to 0.8 M NaCl in R buffer. The peak fractions were collected and further fractionated by gel filtration chromatography (Superdex 200 Increase 10/300 GL, GE Healthcare) in R buffer containing 1 M NaCl. The peak fractions were pooled and dialyzed against R buffer containing 100 mM NaCl. The sample was concentrated by ultrafiltration (Amicon YM-10).

*Escherichia coli* exonuclease I, exonuclease III, T4 DNA polymerase (TaKaRa Bio), AcTEV protease (Thermo Fisher Scientific) and PreScission protease (GE Healthcare) were purchased from the indicated manufacturers.

#### DNA substrates Used for the *in vitr*o DNA Capture Assays

Covalently closed circular plasmid DNA (ccc) of pBluescript II KS (3.0 kb), pSKsxAS (4.3 kb), pKSII-E1 (7.8 kb) and pKSI-E2 (7.8 kb) were prepared using the alkaline lysis method, followed by equilibrium centrifugation in a cesium chloride/ethidium bromide gradient. Relaxed circular (rc) and nicked circular (nc) DNAs were obtained by treating cccDNA with *E. coli* topoisomerase I, Nb.*Bss*SI (pBleuscript KSII) or Nt. *Bsp*QI (pSKsxAS) nicking enzyme, respectively (New England BioLabs). Circular single-stranded (css) DNA of pSKsxAS was prepared with M13KO7 helper phage in accordance to a published protocol (Murayama and Iwasaki, 2011). ΦX174 viron DNA (cssDNA) was purchased (New England BioLabs). Oligo-annealed cssDNA substrate was prepared by annealing of pSKsxAS cssDNA with oligonucleotides 766, 787, 793 and 794 in annealing buffer (50 mM Tris-HCl (pH 7.5), 100 mM KCl and 5 mM MgCl_2_) in a linear temparature gradient of 95 to 4°C (2°C/min). Surplus oligos were removed by gel filtration using S-400 MicroSpin columns (GE Healthcare). Immobilized, closed topology double-stranded (ds) DNA was prepared by PCR amplification of 3.0 kb linear dsDNA with a pair of 5′-biotinylated DNA primers 759 and 760 using pKSII-E2 as the template, followed by immobilization on streptavidin conjugated magnetic beads, essentially as described (Onn and Koshland, 2011). Immobilized cssDNA substrate was prepared by heat-annealing of a 5′-biotinylated oligonucleotide 767 to pSKsxAS cssDNA followed immobilization on streptavidin conjugated magnetic beads, as previously described (Thermo Fisher Scientific) (Kurokawa et al., 2008). The sequence of oligonucleotides used to prepare the DNA substrates are given in Table S2, on overview of the DNA substrates used in our experiments is contained in Table S3.

#### Antibodies Used for Immnoprecipitation and Western Detection

Mouse monoclonal anti-V5 (Pk, Bio-Rad) was used for immunoprecipitation of the cohesin complex via the Psm3-Pk subunit. Mouse monoclonal anit-HA (12CA5, Sigma-Aldrich), anit-E2a (5E11, Abcam) and anti-polyhistidine (MBL) were used for Rad21-HA, Pds5-E2a, E2a-Wapl and His-Psc3 detection. Rabbit polyclonal anti-Psm1, Rad21 and Mis4 (BioAcademia) were used to detect these subunits. The anti-acetyl Smc3 antibody was a kind gift from K. Shirahige, which was used to detect acetylated Smc3 in budding yeast. A rabbit polyclonal anti-RPA serum (Agrisera) that recognizes all three subunits was used to monitor their overexpression. Anti-Tub1 antibody (YOL1/34, BioRad) that detects yeast α-tubulin was used as a loading control. For western detection, HRP-conjugated anti-mouse IgG, anti-rabbit IgG or anti-Rat IgG (GE Healthcare) were used as secondary antibodies.

#### Second-DNA Capture Experiments

Given concentrations and volumes denote final concentrations. In both protocol 1 and protocol 2 reactions, the standard reaction volume is 16.5 μl, containing 100 nM cohesin, 100 nM Mis4-Ssl3 and 100 nM additional Psc3 in CL buffer (35 mM Tris-HCl pH 7.5, 0.5 mM TCEP, 40 mM NaCl, 1 mM MgCl_2_, 15% (w/v) glycerol and 0.003% (w/v) Tween 20). 8 mM phosphocreatine and 8 U/ml creatine kinase (the ATP regeneration system) were included in the reaction if indicated. For a protocol 1 reaction, the reaction mixture was added to closed topology dsDNA beads (containing 3.3 nM closed dsDNA molecules) on ice. The reactions were started by addition of 0.5 mM ATP and incubation at 32°C for 15 min. Free, pKSII-E1 rcDNA (1.3 nM) or pSKsxAS cssDNA (3.3 nM) was added to the reaction and further incubated at 32°C for 5 min. A protocol 1B reaction started as above except that 250 nM cohesin, 125 nM Mis4-Ssl3 and 125 nM additional Psc3 were used. After the first incubation for 30 min, the beads were washed once with CWT buffer (35 mM Tris-HCl pH 7.5, 0.5 mM TCEP, 500 mM NaCl and 0.003% (w/v) Tween 20) and twice with CL buffer. The beads were then resuspended in 13.5 μl CL buffer containing 0.5 mM ATP. Second-DNA capture was initiated by addition of 100 nM Mis4-Ssl3, 100 nM Psc3 and pSKsxAS cssDNA (3.3 nM) and further incubated at 32°C for 5 min. For protocol 2 reactions, proteins were initially incubated with free, pKSII-E1 rcDNA (1.3 nM) or phiX174 cssDNA (3.3 nM) in the presence of 0.5 mM ATP at 32°C for 15 min. The reaction was then transferred onto pSKsxAS cssDNA beads (2.3 nM cssDNA molecules) and further incubated for 5 min. Both type of reactions were terminated by addition of 750 μl of ice-chilled CW1 buffer (35 mM Tris-HCl pH 7.5, 0.5 mM TCEP, 800 mM NaCl, 0.35% (w/v) Triton X-100) and rocked at 4°C for 10 min. The recovered beads were washed three times with CW1 buffer and once with CW2 buffer (35 mM Tris-HCl pH 7.5, 0.5 mM TCEP, 100 mM NaCl and 0.1% (w/v) Triton X-100). The captured DNA was then eluted in 15 μl of elution buffer (10 mM Tris-HCl pH 7.5, 1 mM EDTA, 50 mM NaCl, 0.75% SDS and 1 mg/ml protease K) by incubating at 37°C for 20 min. The recovered DNA was separated by 1% agarose gel electrophoresis in TAE buffer at 20°C (room temperature) at 3.5 V/cm for 80 min and stained with SYBR gold (Thermo Fisher). Gel images were captured using an ImageQuant LAS-4000 Mini gel documentation system (Fuji Film) and band intensities were quantified with MultiGauge software (Fuji Film).

In experiments including ssDNA to dsDNA conversion, second-DNA capture reactions were performed as described above using pSKsxAS cssDNA to which 4 short oligonucleotides were annealed (see DNA substrates). Following second-DNA capture, DNA beads were washed twice with CW1 buffer then twice with by T4 polymerase buffer (20 mM Tris-HCl pH 7.5, 0.5 mM TCEP, 40 mM NaCl, 10 mM MgCl_2_ and 0.01% (w/v) Triton X-100). The beads were incubated with T4 DNA polymerase (0.25 U/μl, TaKaRa Bio) at 32°C for 20 min in T4 polymerase buffer containing 0.5 mM dNTPs (TaKaRa). The beads were now washed once with CW1 buffer and twice with RE buffer (35 mM Tris-HCl pH 7.5, 0.5 mM TCEP, 100 mM NaCl, 10 mM MgCl_2_ and 0.1% Triton X-100). For restriction digestion, the recovered beads were resuspended in 10 μl of RE buffer containing PstI (1 U/μl, Nippon Gene) or BglII (1 U/μl, TaKaRa Bio) and incubated at 18°C for 1 h. The NaCl concentration was adjusted to 500 mM in 15 μl and incubated on ice for 15 min, before the supernatant and beads fractions were analyzed by 1% agarose gel electrophoresis as described above. For TEV protease cleavage, the beads incubated with T4 DNA polymerase were washed once with CW1 buffer and twice in TEV buffer (35 mM Tris-HCl pH 7.5, 0.5 mM TCEP, 150 mM NaCl and 0.1% (w/v) Triton X-100). The beads were resuspended in TEV buffer containing TEV protease (0.3 U/μl, ThermoFisher) and incubated 18°C for 1.5 h. The NaCl concentration was adjusted to 500 mM in 15 μl and incubated on ice for 15 min. The resultant supernatant and beads fractions were analyzed by 1% agarose gel electrophoresis as described above. The TEV protease cleavage using protocol 2, followed by ssDNA to dsDNA conversion, was also carried out as described above. The resultant beads were washed twice with CW1 buffer, then twice in TEV buffer II (35 mM Tris-HCl pH 7.5, 0.5 mM TCEP, 50 mM NaCl, 10 mM MgCl_2_ and 0.1% (w/v) Triton X-100). The beads were resuspended in TEV buffer II containing TEV protease (0.3 U/μl, ThermoFisher) and incubated at 16°C for 1.5 h. The beads were washed three times with CW1 buffer and once with CW2 buffer. The beads-bound DNA was eluted and analyzed by 1% agarose gel electrophoresis as described above.

For the NaCl-EDTA stability assay, second-DNA capture followed by ssDNA to dsDNA conversion was carried out as described above. The resultant beads were washed twice with CW1 buffer, then with ST buffer (35 mM Tris-HCl pH 7.5, 0.5 mM TCEP, 100 mM NaCl and 0.1% (w/v) Triton X-100). The beads were resuspended in 10 μl of ST buffer containing 10 mM EDTA and incubated at 32°C for 15 min. The NaCl concentration was adjusted to 500 mM in 15 μl and incubated on ice for 15 min, before the supernatant and beads fractions were analyzed by 1% agarose gel electrophoresis as described above.

#### Cohesin DNA Loading Assay

Standard reactions (15 μl final volume) were performed as described (Murayama and Uhlmann, 2014). Cohesin (100 nM), Mis4-Ssl3 (100 nM), Pds5-Wapl (100 nM), additional Psc3 (100 nM) and rc or cssDNA were mixed on ice in CL buffer. pBluescript KSII rcDNA or pSKsxAS cssDNA were used as dsDNA or ssDNA substrate, respectively. The reactions were initiated by addition of ATP (0.5 mM) and incubated at 32°C for 30 min, if not otherwise indicated. The reactions were terminated by addition of 500 μl of ice-chilled CW1 buffer and incubated for 5 min on ice. Anti-Pk (V5, Bio-Rad) bound to protein A conjugated magnetic beads (Thermo Fisher) were added to the terminated reactions and rocked at 4°C overnight (∼15 h). The beads were three times washed with CW1 buffer and once with CW2 buffer. The cohesin-bound DNA was eluted in 15 μl of elution buffer by incubating at 50°C for 20 min. The recovered DNA was separated by 1% agarose gel electrophoresis in TAE buffer at 20°C (room temperature) at 3.5 V/cm for 60 min and stained with SYBR gold.

For the NaCl-EDTA chase assay, cohesin loading reactions (15 μl) were carried out as described above. 15 μl of CL buffer containing 260 mM NaCl (150 mM, final) and 20 mM EDTA (10 mM final), preincubated at 32°C, were added to the reactions and further incubated for 15 min. The DNA-bound cohesin was retrieved and analyzed as described above.

In experiments including ssDNA to dsDNA conversion followed by linearization, the loading reactions were performed as described with pSKsxAS cssDNA annealed with 4 short oligonucleotides and the cohesin-cssDNA complexes were retrieved by immunoprecipitation. The resultant beads were washed, incubated with T4 DNA polymerase, followed by PstI cleavage, using the same procedure as described above in the second-DNA capture section.

For dsDNA to ssDNA conversion of cohesin-bound ncDNA, cohesin loading reactions were carried out with pBluescript ncDNA as described above. The cohesin-ncDNA bound beads were washed twice with CW1 buffer then twice with exoIII buffer (25 mM Tris-HCl pH 7.5, 1 mM TCEP, 100 mM NaCl and 0.01% (w/v) Triton X-100). The beads were incubated in 10 μl of exoIII buffer containing *E. coli* exonuclease III (5 U/μl, TaKaRa Bio) at 30°C for 20 min. EDTA and NaCl concentrations were adjusted to 10 and 150 mM, respectively, and incubation continued at 30°C for 15 min. The NaCl concentration was now adjusted to 500 mM in 15 μl and incubated on ice for 15 min, before the supernatant and beads fractions were analyzed by 1% agarose gel electrophoresis as described above. To confirm circular integrity of cohesin-released ssDNA after exonuclease III treatment, the supernatant fraction was passed through a G-25 gel filtration spin column, equilibrated with exoI buffer (67 mM Glycine-KOH pH 9.5, 1 mM DTT and 6.7 mM MgCl_2_). The recovered DNA was incubated with *E. coli* exonuclease I (0.5 U/ μl, TaKaRa Bio) in 10 μl of exoI buffer at 30°C for 15 min. The reactions were terminated by addition of 2.5 μl of stop solution (1% SDS, 10 mM EDTA and 4 mg/ml protease K), incubated at 37°C for 20 min, and analyzed by 1% agarose gel electrophoresis.

#### ATPase Assay

Cohesin (150 nM) and Mis4-Ssl3 (100 nM) were mixed with pBluescript rcDNA or ΦX174 cssDNA in CL buffer (15 μl in final volume). The reactions were initiated by addition of 0.25 mM ATP, spiked with [γ-^32^P]-ATP, and incubated at 32°C. Aliquots (1.5 μl) were taken after 0, 15, 30 and 60 min and terminated by addition of 4.5 μl of 0.5 M EDTA pH 8.0. 1 μl of the reaction mixture was spotted on polyethylenimine cellulose F sheets (Merck) and the products were separated by thin layer chromatography, developed with 400 mM LiCl in 1 M formic acid. Plates were analyzed and ATP hydrolysis was quantified using a Phosphorimager (GE Healthcare).

#### Electrophoretic Mobility Shift Assay

Cohesin was mixed with circular ssDNA (pBluescript SKII, 1.67 nM) or ncDNA (pBluescript KSII, 1.67 nM) at increasing concentrations in CL buffer and incubated at 32°C for 15 min. Samples were analyzed by 0.8% agarose gel electrophoresis in TAE buffer at 20°C (room temperature) at 3.5 V/cm for 120 min. DNA molecules were stained with SYBR gold (Thermo Fisher) and gel images were captured and analyzed as described above.

WT or Rfa1^G78E^ mutant RPA complexes were mixed with 5′ tetramethylrhodamine (TAMRA)-conjugated oligo(dT)_54_ (100 nM) at increasing concentrations in CL buffer and incubated at 32°C for 15 min. Samples were analyzed by electrophoresis through a 10% acrylamide gel in TAE buffer at 20°C (room temperature) at 5 V/cm for 90 min.

#### Construction of the Budding Yeast *rfa1*^*G77E*^ Strain

The *rfa*^*G77E*^ strains were generated by gene replacement. pScRPA^G77E^ 3Pk::kanMX6 was digested with *Xba*I and resultant DNA fragments were transformed into budding yeast (Y141, see Table S1). G418 resistant cells were selected and gene replacement was confirmed by DNA sequencing. The *rfa*^*G77E*^ allele was then introduced into strains harboring mutations in cohesion establishment factors by genetic crossing. Two plasmids expressing the three subunits of budding yeast RPA under control of the bidirectional *GAL1-10* promoter (Yeeles et al., 2015) were integrated into the budding yeast genome to achieve inducible RPA overexpression. Single copy integration of both plasmids was confirmed by PCR.

#### *In Vivo* Sister Chromatid Cohesion Assay

Budding yeast cultures were arrested in G1 by pheromone α-factor treatment (0.4 μg/ml) for 2 hours. Cells were washed with fresh medium lacking α-factor and released from the α-factor block for synchronous progression through the cell cycle and arrest in mitosis in medium containing 5 μg/ml nocodazole for 120 min. Alternatively, cells were released from α-factor block into medium containing 0.2 M hydroxyurea for 60 min, then released to progress through S phase and into nocodazole-imposed mitotic arrest. *MET-CDC20* cells were grown in medium lacking methionine and shifted to medium containing 8 mM methionine following α-factor release. Analysis of sister chromatid cohesion was performed as previously (Michaelis et al., 1997). To visualize the GFP-marked chromosomal loci, cell were fixed in ice-cold ethanol. Cells were then sonicated and immobilized on a slide covered with a thin layer of 2% agarose. The sister chromatid cohesion state was observed using an Axioplan 2 imaging microscope (Zeiss) equipped with a 100x (NA = 1.45) Plan-Neofluar objective.

#### Western Blotting for Budding Yeast Acetyl Smc3 and RPA Detection

Budding yeast cultures were synchronized as described above. Cells were taken at indicated time points and protein extracts were prepared from TCA fixed cells (Foiani et al., 1994), separated by SDS-PAGE and blotted on a nitrocellulose membrane. The membrane was blocked in PBS buffer containing 0.05% (w/v) of tween 20 and 5% (w/v) of skimmed milk powder. To detect budding yeast acetyl Smc3 and RPA, a mouse monoclonal anti-acetyl Smc3 antibody (a gift from K. Shirahige) and anti-RPA antiserum (Agrisera) were used, respectively. Tubulin served as a loading control and was detected using an anti-tubulin antibody (Bio-Rad). Peroxidase-coupled secondary antibodies were used.

### Quantification and Statistical Analysis

#### Second-DNA Capture and Cohesin Loading Experiments

Recovered DNA was separated by agarose gel electrophoresis. After staining with SYBR gold, recovered DNA was quantified by fluorescent-imaging using an ImageQuant LAS-4000 Mini gel documentation system using excitation at 460 nm with Y515 filter setting (Fujifilm). The DNA band intensities were measured using MultiGauge software (version 3.2, Fujifilm) and the retrieved DNA was quantified as a percentage of DNA input, loaded alongside. The graphs depict means and the error bars represent standard deviations from at least three independent experiments, if not otherwise indicated.

#### ATPase Assay

Reaction products were separated by thin layer chromatography and quantified using a Phosphorimager (Typhoon FLA9500, GE Healthcare) and ImageQuant TL software (GE Healthcare). ATP hydrolysis at each time points was calculated from the ratio of inorganic monophosphate and ATP and the velocities were calculated from the observed rates of ATP hydrolysis.

#### *In vivo* Sister Chromatid Cohesion Assay

Data are expressed as percentage of cells displaying visibly split or separated GFP-marked loci of the total cell count in each sample. For each condition, at least 100 cells were scored and each experiment was repeated three times. Means and standard deviations are reported for each condition.

#### Western blotting

The blotted proteins were detected using ECL reagents. Signals were quantified using an Amersham Imager 600 (GE Healthcare) and in addition detected on films for visualization.

### Data and Software Availability

Data have been deposited with the Mendeley Database and are available at https://data.mendeley.com/datasets/wzmx278bj7/draft?a=60051c6f-7d50-42b8-abd8-67026ca46c18
