# Supplementary material for: Establishment of DNA-DNA Interactions by the Cohesin Ring
Source: Cell. 2018 Jan 25;172(3):465–477.e15. doi: 10.1016/j.cell.2017.12.021 (PMC5786502; doi:10.1016/j.cell.2017.12.021)
Supplement: Document S1. Tables S1-S3 [file mmc1.pdf]

Cell, Volume *172*

Supplemental Information

Establishment of DNA-DNA Interactions

by the Cohesin Ring

Yasuto Murayama, Catarina P. Samora, Yumiko Kurokawa, Hiroshi Iwasaki, and Frank Uhlmann

Table S1. Fission and Budding Yeast Strains Used in this Study

strain name	species	genotype	purpose
Y4483	*S. pombe*	*h- pMis4-PA (LEU2), pSsl3(ura4+), lue1-32 ura4-D18*	Mis4-Ssl3 purification
Y4711	*S. pombe*	*h- pMis4ΔN-PA (LEU2), lue1-32 ura4-D18*	Mis4 *Δ*N purification
Y4443	*S. cerevisiae*	*MAT*a *URA3::pGAL1-psm3-3Pk-7his / pGAL10-psm1 LEU2::pGAL1-rad21-HA-pps-protein A / pGAL10-7his-psc3, pep4Δ::HIS3*	WT Cohesin purification
Y4455	*S. cerevisiae*	*MAT*a *URA3::pGAL1-psm3^{E1128Q} -3Pk-7his / pGAL10-psm1^{E1161Q} LEU2::pGAL1-rad21-HA-pps-protein A / pGAL10-7his-psc3, pep4Δ::HIS3*	1B3B Cohesin purification
Y4449	*S. cerevisiae*	*MAT*a *URA3::pGAL1-psm3-3Pk-7his / pGAL10-psm1 LEU2::pGAL1-rad21TEV-HA-pps-protein A / pGAL10-7his-psc3, pep4Δ::HIS3*	21TEV Cohesin purification
Y4838	*S. cerevisiae*	*MAT*a *URA3::pGAL1-psm3^{K106Q}-3Pk-7his / pGAL10-psm1 LEU2::pGAL1-rad21-HA-pps-protein A / pGAL10-7his-psc3 pep4Δ ::HIS3*	KQ Cohesin purification
Y4755	*S. cerevisiae*	*MAT*a *URA3::pGAL1-psm3^{K105Q,K106Q}-3Pk-7his / pGAL10-psm1 LEU2::pGAL1-rad21-HA-pps-protein A / pGAL10-7his-psc3 pep4Δ::HIS3*	KKQQ Cohesin purification
Y4743	*S. cerevisiae*	*MAT*a *TRP1::pGAL1-pds5-E2a-pps-proteinA, pep4Δ::HIS3*	Pds5 purification
Y141	*S. cerevisiae*	wild type strain (*MAT*a *ura3-52, ade2-1, trp1-1, can1-100, leu2-3,112, his3-11,15*)	Figure S6A
Y5273	*S. cerevisiae*	*MAT*a *rfa1G77E-3HA:: kanMX*	Figure S6A
Y168	*S. cerevisiae*	*MAT*a *scc1-73*	Figure S6A
Y4323	*S. cerevisiae*	*MAT*a *ctf4Δ::TRP1*	Figure S6A, C
K7100	*S. cerevisiae*	*MAT*a *URA3::tetOs HIS3::tetR-GFP*	Figure 7A, B, C, S6B, C, E, F
Y5333	*S. cerevisiae*	*MAT*a *URA3::tetOs HIS3::tetR-GFP rfa1^{G77E}-3HA::kanMX6*	Figure7A, B, S6B, C, E
Y3853	*S. cerevisiae*	*MAT*a *ura3::3URA3 tetO112, his3::HIS3 tetR-GFP ctf18Δ::TRP1*	Figure 7A, B, S6B, C
Y5335	*S. cerevisiae*	*MAT*a *ura3::3URA3 tetO112, his3::HIS3 tetR-GFP ctf18Δ::TRP1 rfa1^{G77E}-3HA::kanMX6*	Figure 7A, B, S6B, C
Y5479	*S. cerevisiae*	*MAT*a *ura3::3URA3 tetOx200, leu2::LEU2 tetR-GFP HIS::Gal1-10 CBP-Tev-RFA1+Gal4 TRP::Gal1-10 RFA2+RFA3*	Figure 7A, S6B
Y5480	*S. cerevisiae*	*MAT*a *ura3::3URA3 tetOx200, leu2::LEU2 tetR-GFP HIS::Gal1-10 CBP-Tev-RFA1+Gal4 TRP::Gal1-10 RFA2+RFA3 Δctf18::KAN*	Figure 7A, S6B
Y5271	*S. cerevisiae*	*MAT*a *MET-CDC20::URA3 tetR-GFP::LEU2 CENV::tetO2x224::URA Spc42-tdTomato::NAT*	Figure S6D
Y5341	*S. cerevisiae*	*MAT*a *MET-CDC20::URA3 tetR-GFP::LEU2 CENV::tetO2x224::URA Spc42-tdTomato::NAT chl4Δ::TRP1*	Figure S6D

Y5342	*S. cerevisiae*	*MAT*a *MET-CDC20::URA3 tetR-GFP::LEU2 CENV::tetO2x224::URA Spc42-tdTomato::NAT chl4Δ::TRP1 rfa1*^{G77E}*-3HA::kanMX6*	Figure S6D
Y3856	*S. cerevisiae*	*MAT*a *ura3::3URA3 tetO112, his3::HIS3 tetR-GFP chl1Δ::TRP1*	Figure S6E, F
Y5334	*S. cerevisiae*	*MAT*a *ura3::3URA3 tetO112, his3::HIS3 tetR-GFP chl1Δ::TRP1 rfa1*^{G77E}*-3HA::kanMX6*	Figure S6E, F
Y3569	*S. cerevisiae*	*MAT*a *ura3::3URA3 tetO112, his3::HIS3 tetR-GFP eco1-1::TRP1*	Figure 7C, S6E
Y5338	*S. cerevisiae*	*MAT*a *ura3::3URA3 tetO112, his3::HIS3 tetR-GFP eco1-1::TRP1 rfa1*^{G77E}*-3HA::kanMX6*	Figure S6E
Y180	*S. cerevisiae*	*MAT*a *ura3::3URA3 tetO112, leu2::LEU2 tetR-GFP scc2-4*	Figure 7C

All budding yeast strains are W303 background (*ura3-52, ade2-1, trp1-1, can1-100, leu2-3,112, his3-11,15*)

PA: Protein A, 3Pk: 3xV5 tag, 7his: 7 histidine tag, pps: PreScission protease site, 3HA: 3xHA tag

Table S2. Sequence of the nucleotides used for *in vitro* assays (related to Figure 1-5,7 and S1-4, S6)

name	sequence (5' - 3')
766	5'-TCGCCACTGGCAGCAGCCACTGGTAACAGGATTA-3'
767	5'-[bioTEG]GGTCTGACAGTTACCAATGCTTAATCAGTGAGGCACCTATCTCAGCGATCTGTCTATTTCGTTCATCCAT AGTTGCCTGACTCCCCGTCGTGTAGATAAC-3’
787	5’-AATCGGCGTTCCTGTAAAAGGAAGCACTCTTCTGCAGCCCGGGGGATCCACTAGAGCGGCCGCCAC-3’
793	5’-GTTGAAAAGTTCGTTCCTTTCTCCATGGAAATTGCAGTGATGGTAGTACGCAGTTTAGACGG-3’
794	5’-CTAAATTGTAAGCGTTAATATTTTGTTAAAATTCGCGTTAAATTTTTGTTAAATCAGCTC-3’
Iwa971	5’-[bioTEG]ATAAGGCCACGTATTTTGCAAGCTATTTAACTGGCGGCGATTGCGTACCCGACGACCAAAATTAGGGTC AACGCTACCTGTAGGAAGTGTCCGCATAAAG-3'
747	5'-CGCGCAATTAACCCTCACTAAAGG-3'
759	5'-[bioON]CGCGCAATTAACCCTCACTAAAGG-3'
760	5'-[bioON]CCCTTTCGGTTTTTTATGAAATTATGATGG-3'
dT54	5’-[TAMRA]TTTTTTTTTTTTTTTTTTTTTTTTTTTTTTTTTTTTTTTTTTTTTTTTTTTTTT-3’

Table S3. DNA substrates used for *in vitro* assays (related to Figure 1-5,7 and S1-4, S6)

structure	origin	strand	length	preparation	oligos (annealing / PCR)	assay
css	pBluescript SKII	ss	3.0 kb			electrophoretic mobility shift assay
rc	pBluescript KSII	ds	3.0 kb			initial DNA loading
nc	pBluescript KSII	ds	3.0 kb			initial DNA loading followed by dsDNA to ssDNA conversion, electrophoretic mobility shift assay
css	pSKsxAS	ss	4.3 kb			initial DNA loading, 2nd DNA capture protocol 1
css + OL	pSKsxAS	ss	4.3 kb	annealing	766, 787, 793, 794	initial DNA loading, 2nd DNA capture protocol 1
nc	pSKsxAS	ds	4.3 kb			2nd DNA capture protocol 2, dsDNA competition assay
closed (beads)	pKSII-E2	ds	3.0 kb	PCR and beads attachment	759, 760	2nd DNA capture protocol 1
linear (beads)	pKSII-E2	ds	3.0 kb	PCR and beads attachment	747, 760	2nd DNA capture protocol 1
rc	pKSII-E1	ds	7.8 kb			2nd DNA capture protocol 1 and 2
css	phiX174 viron	ss	5.4 kb			2nd DNA capture protocol 2
css (beads)	pSKsxAS	ss	4.3 kb	annealing and beads attachment	767	2nd DNA capture protocol 2
css + OL (beads)	pSKsxAS	ss	4.3 kb	annealing and beads attachment	767, 787, 793, 794	2nd DNA capture protocol 2
css (beads)	phiX174 viron	ss	5.4 kb	annealing and beads attachment	Iwa971	2nd DNA capture protocol 2

rc: relaxed circular dsDNA, nc: nicked circular dsDNA, css; circular ssDNA, OL: DNA oligo.
